# In Vitro and In Vivo Evaluation of a Composite Electrospun Matrix as a Dermal Scaffold: Cell Behavior and Full-Thickness Wound Repair

**DOI:** 10.3390/jfb17080375

**Published:** 2026-08-01

**Authors:** Chenhong Wang, Christopher Bibbo, Mark Suski, Xianghua Xu, Sean Chen

**Affiliations:** 1 Kreate Medical Inc., 2300 Lakeview Parkway, Suite 700, Alpharetta, GA 30009, USA; 2 Department of Orthopaedic Surgery, Temple University Lewis Katz School of Medicine, 3401 N. Broad Street, 5th Floor, Philadelphia, PA 19140, USA; 3 Los Robles Regional Medical Center, 227 West Janss Rd., Ste 320, Thousand Oaks, CA 91360, USA

**Keywords:** electrospun matrix, dermal scaffold, absorbable wound matrix, staged absorption, full-thickness wound repair

## Abstract

An effective dermal scaffold is expected to provide a three-dimensional porous architecture that supports fibroblast adhesion, infiltration, and proliferation; evolve structurally to meet the progressive physiological needs of the entire healing process; and absorb without adverse tissue response during tissue repair. This study evaluates a fully synthetic absorbable composite electrospun matrix comprising three polymer components within a single fiber network, each contributing a distinct yet complementary function. Poloxamer 188 provides immediate wettability and conformability; PLGA undergoes progressive hydrolytic degradation over days, enlarging pore dimensions through fiber cleavage; and polydioxanone, as the slowest-degrading component, maintains the fiber network throughout this structural remodeling process. The matrix achieved instantaneous wetting and 91% maximum pore-equivalent diameter enlargement within 7 days, creating a microenvironment associated with progressive cellular accommodation. In vitro, fibroblasts adhered, remained fully viable, and exhibited Day-1 spreading on the 7-day pre-degraded matrices approaching that observed at Day 3 on fresh matrices, consistent with the evolving pore architecture facilitating initial cellular accommodation. In a splinted rat full-thickness wound model, the matrix accelerated wound-area reduction versus the control (Day 14: *p* < 0.001; Day 21: *p* < 0.01), was macroscopically undetectable by Day 14, and elicited no adverse foreign-body reaction. Quantitative collagen area fraction analysis (Masson’s trichrome) revealed significantly greater collagen content in the scaffold group at Day 7 (*p* < 0.05) and Day 28 (*p* < 0.01) relative to the control, suggesting that the composite matrix provides favorable conditions for collagen accumulation that persists into the remodeling phase. These findings demonstrate that the matrix functions as an effective dermal scaffold by providing an evolving pore architecture that supports fibroblast adhesion and accommodation, accelerating full-thickness repair throughout the healing trajectory, and undergoing safe absorption without adverse reactions, thereby highlighting its promising translational potential for clinical wound management.

## 1. Introduction

Full-thickness skin defects resulting from trauma, burns, and chronic ulcers eliminate the dermal regenerative template required for organized fibroblast repopulation and re-epithelialization, necessitating exogenous scaffolds that restore the lost structural framework during granulation tissue formation [1,2,3]. An effective dermal scaffold is expected to satisfy three functional criteria simultaneously: (i) immediate wettability and wound-bed conformance upon application, enabling intimate scaffold–tissue contact without pre-conditioning; (ii) an interconnected pore architecture that not only provides sufficient initial dimension for cell attachment but also evolves to progressively support fibroblast adhesion, infiltration, and proliferation in three dimensions, rather than restricting cellular engagement to the material surface; and (iii) sustained mechanical coherence throughout the critical proliferative phase, followed by complete absorption during tissue repair, leaving a residue-free wound bed that permits unimpeded endogenous remodeling or, if clinically indicated, repeat intervention [4,5,6,7].

In conventional single-polymer electrospun systems, criteria (ii) and (iii) are governed by a constraining structural interdependence: the mechanical coherence of an electrospun network is predicated upon fiber packing density and the continuity of inter-fiber junctions [8,9,10], yet attaining the pore dimensions requisite for three-dimensional cellular infiltration necessitates the disruption of precisely these microstructural elements—rendering simultaneous fulfillment of both criteria unachievable within a single-component architecture. Resolving these conflicting structural demands within a single construct represents a central design challenge in dermal scaffold engineering. Existing scaffold approaches address subsets of these criteria while compromising others. Biologic matrices derived from decellularized dermis closely mimic native extracellular matrix (ECM) composition and provide favorable cell-recognition motifs; however, they exhibit lot-to-lot variability, limited control over pore architecture, and susceptibility to premature enzymatic degradation in protease-rich wound environments—limitations that collectively hinder the consistent fulfillment of criteria (ii) and (iii) [11]. An additional limitation intrinsic to synthetic electrospun scaffolds pertains to criterion (i): the aliphatic polyesters predominantly employed in electrospinning—PLGA, PCL, and PDO—are inherently hydrophobic (water contact angles typically >110°) [12,13]. Upon wound-bed contact, entrapped air within inter-fiber interstices precludes the spontaneous capillary imbibition necessary for immediate scaffold–tissue apposition. Overcoming this wetting barrier in practice requires pre-conditioning protocols such as graded ethanol priming or plasma surface activation [12], adding procedural complexity and performance variability. Additionally, in the absence of fluid-mediated plasticization, the dry fiber network remains mechanically non-conformable to irregular wound-bed geometries—a limitation unaddressed by pore-enlargement strategies alone. Electrospun synthetic matrices offer reproducible fabrication and compositional tunability, yet the dense fiber packing intrinsic to the process restricts inter-fiber pore dimensions to a few to tens of micrometers [14,15]—well below the ~100–200 µm pore range demonstrated to support active three-dimensional fibroblast infiltration and ECM deposition in electrospun scaffolds [16,17]—thereby failing criterion (ii). Post-fabrication strategies intended to enlarge pores, including mechanical perforation, sacrificial porogen leaching, and ultrasonication, compromise fiber continuity, introduce processing complexity, or yield static architectures whose porosity is permanently fixed at the time of fabrication and cannot adapt to the evolving demands of tissue repair [10,17,18,19]. A more fundamental limitation underlies all such approaches: because wound healing proceeds through temporally distinct phases—each placing different and evolving structural demands on the scaffold—a pore architecture optimized for one phase is inherently suboptimal for another [20,21]. Consequently, no existing single-polymer electrospun system has been demonstrated to simultaneously satisfy all three criteria across the healing trajectory.

The composite electrospun matrix described herein was designed to resolve this constraint by assigning each functional criterion to a dedicated polymer component whose absorption timescale is matched to its designated biological role—all within a single co-electrospun fiber network. Poloxamer 188 (P188), an amphiphilic triblock copolymer with predominant hydrophilic character, dissolves within seconds of aqueous contact, rendering the otherwise hydrophobic fiber network immediately wettable and, through the plasticizing effect of hydration, conformable to the wound-bed contour—fulfilling criterion (i). Poly(lactic-co-glycolic acid) (PLGA), selected for its rapid hydrolytic kinetics (on the order of days), undergoes progressive fiber cleavage and junction disruption within the first week [22], enlarging initial inter-fiber voids into an increasingly open, interconnected pore architecture within the ~100–200 µm range reported to support dermal fibroblast infiltration [16]—fulfilling criterion (ii). Polydioxanone (PDO), with a reported in vivo strength-retention half-life of 4–6 weeks and complete absorption within approximately six months [23,24,25], maintains mechanical coherence of the remaining fiber network throughout the proliferative phase, well beyond the point at which the faster-absorbing PLGA component has been consumed, ensuring structural continuity until absorption yields a residue-free wound bed—fulfilling criterion (iii). This temporal hierarchy—seconds, days, and weeks to months—converts matrix absorption from passive material loss into a staged structural adaptation matched to wound biology. Importantly, this modular design principle extends beyond the specific polymer combination employed here: absorption timescale, rather than polymer identity, serves as the governing design variable [7,25]. While specific polymer mass ratios are proprietary, the functional consequences of the composite architecture are fully characterized in the sections that follow.

The present study evaluates whether this staged-absorption composite electrospun matrix (hereafter termed the Fibrous Matrix) can function as a dermal scaffold by satisfying the three functional criteria defined above—specifically, whether its evolving pore architecture supports fibroblast adhesion, infiltration, and three-dimensional spreading (hereafter termed cellular accommodation) during matrix residence, and whether it undergoes timely absorption without adverse tissue response while enhancing wound repair relative to inert coverage. Three complementary levels of evidence were generated to address this question. Physicochemical characterization assessed wettability, pore evolution kinetics, and degradation behavior at the material level. In vitro morphometric analysis of L929 murine and BJ human fibroblasts examined whether progressive pore enlargement—rather than culture duration alone—governs cellular accommodation. In vivo evaluation employed a splinted rat full-thickness excisional wound model (Ø 20 mm; *n* = 40; 28-day follow-up) to assess wound-area reduction, matrix absorption, biocompatibility (ISO 10993-6 [26]), and collagen deposition relative to a standard-of-care control.

## 2. Materials and Methods

### 2.1. Preparation and Characterization of the Composite Fibrous Matrix

#### 2.1.1. Composite Matrix Fabrication

The composite fibrous matrix was fabricated via electrospinning using a TL Series Electrospinning System (Tongli Tech Co., Ltd., Shenzhen, China). The composite matrix comprises different types of composite fibers containing varying combinations of the constituent polymers (PDO, PLGA, and P188), with P188 content below 1% (*w*/*w*) of the total matrix mass. Final matrix thickness is approximately 200 µm. Electrospinning was conducted at room temperature (chamber relative humidity 30–60%) with a solution flow rate of 3–15 mL/h, an applied voltage of 12–25 kV, and a tip-to-collector distance of 10–30 cm. A schematic of the electrospinning configuration is provided in Figure S1. The resulting non-woven fibrous matrix was subjected to a validated drying process to remove residual solvents; final residual solvent levels were confirmed to meet extractable and leachable requirements for Class II medical devices per ISO 10993-18:2020 [27] prior to sterilization. All samples were sterilized by electron-beam irradiation and stored in sealed packaging until use. Test specimens were used directly from packaging without further conditioning. While specific polymer mass ratios and detailed process parameters are proprietary, the composite architecture and its functional properties are fully characterized in Section 2.1.2, Section 2.1.3, Section 2.1.4 and Section 3.1 [22,23,25]. Researchers seeking additional compositional or process details for collaborative purposes are invited to contact the corresponding author.

#### 2.1.2. Wettability, Fluid Uptake, and Mechanical Characterization

Wettability, fluid uptake, and tensile properties were characterized by the following methods.

Immersion wetting. A dry matrix sheet was placed onto the surface of phosphate-buffered saline (PBS, pH 7.4) at room temperature. The wetting front progression was recorded (DJI Osmo Pocket 3, Shenzhen, China; 30 fps) at 0, 1, 3, 4, and 5 s.

Drop absorption. Deionized-water droplets (3–5 µL) were deposited onto the dry matrix surface via micropipette with the specimen positioned horizontally. The absorption process was recorded by smartphone video (30 fps; Video S1). The drop absorption measurement principle—depositing a controlled-volume liquid droplet onto a specimen surface and recording the time to complete absorption (loss of surface specular reflection)—is analogous to the textile absorbency standard AATCC TM79 [28].

Contact angle measurement. Static contact angle was measured using a contact angle goniometer (Kyowa Interface Science Co., Ltd., Saitama, Japan). Deionized-water droplets (~3 µL) were dispensed onto the matrix surface; owing to rapid liquid penetration, sequential frames were captured at *t* = 0, 15.5, 31, and 46 ms immediately post-contact rather than following standard equilibrium protocols [29].

Fluid absorption capacity. Fluid absorption capacity was evaluated in accordance with YY/T 0471.1-2004 (IDT EN 13726-1:2002; current edition: EN 13726:2023) [30,31]. Matrix specimens (5 × 5 cm, *n* = 10) were immersed in simulated wound exudate (142 mmol/L Na^+^, 2.5 mmol/L Ca^2+^) at 37 ± 1 °C for 30 min, drained vertically for 30 s, and reweighed. Fluid absorption was calculated as

Fluid absorption (%) = (*W*_2_ − *W*_1_)/*W*_1_ × 100%

where *W*_1_ denotes initial dry mass and *W*_2_ denotes mass after immersion and drainage.

Tensile properties. Tensile properties were characterized using an electronic universal testing machine (Model FYW-L, Jinan Fangyuan Test Instrument Co., Ltd., Jinan, China) at a crosshead speed of 100 mm/min with reference to ASTM D882 [32]. Rectangular specimens (50 mm wide × 0.2 mm thickness) were clamped along the short edge; peak tensile load was recorded (*n* = 5 per lot, three independent production lots).

#### 2.1.3. In Vitro Degradation

Matrix degradation was characterized under two complementary conditions addressing distinct experimental objectives.

Structural adaptation in cell-culture medium. To recapitulate the microenvironment experienced by cells during in vitro culture, samples were incubated in complete medium (Dulbecco’s Modified Eagle Medium (DMEM) supplemented with 10% fetal bovine serum (FBS) and 1% penicillin–streptomycin) at 37 °C in a humidified 5% CO_2_ atmosphere (medium-to-matrix ratio of 1 mL per 6 cm^2^). Samples were retrieved at Days 0 (as-fabricated), 5, 7, and 14 for scanning electron microscopy (SEM) morphological characterization and pore metric analysis (Section 2.1.4).

Accelerated enzymatic degradation. To quantify bulk degradation kinetics and simulate the enzymatic environment of wound exudate, samples (1.25 cm × 1.88 cm) were immersed in 20 mL PBS (8.0 g/L NaCl, 0.2 g/L KCl, 3.76 g/L Na_2_HPO_4_·12H_2_O, 0.2 g/L KH_2_PO_4_, pH 7.4) supplemented with 4 g/L lipase at 45 °C [22]. The solution was replaced every 2 days over 28 days (*n* = 3 per time point). At predetermined intervals (Days 0, 4, 8, 12, 16, 20, 24, and 28), samples were retrieved, rinsed three times with distilled water, and dried to constant weight in a vacuum oven (40 °C, 24 h). Mass remaining was calculated as

Mass remaining (%) = W*t*/*W*_0_ × 100%

where *W*_0_ denotes initial dry weight and W*t* denotes dry weight at time point *t*.

#### 2.1.4. SEM and Quantitative Pore Analysis

Matrix morphology was observed by field-emission SEM (Hitachi SU8220, Tokyo, Japan; 5 kV, SE(UL) detector). Samples were sputter-coated with gold (~10 nm, 30 s) prior to imaging at 500× magnification (spatial calibration: 10 px/µm). Quantitative pore analysis was performed using a custom Fiji/ImageJ macro [33,34]: images were filtered (Difference-of-Gaussians), binarized (Otsu-derived global threshold), and processed with morphological closing and opening (one iteration each). Pores < 0.5 µm^2^ were excluded. Metrics extracted included porosity (%), maximum pore-equivalent diameter (EqD = √(4A_max/π)), Connectivity Index (CI = Junctions/(Junctions + Endpoints)), and Fiber Density (total skeleton length/image area, µm/µm^2^). Threshold robustness was verified by repeating analysis at ±5 gray levels; a Robustness Index was calculated as RI = 1 − |ΔMetric(Thr ± 5)|/|ΔMetric(D_7_ − D_0_)|.

### 2.2. In Vitro Cell Behavior Study

#### 2.2.1. Cell Culture

NCTC clone 929 (L929) murine fibroblasts (Procell, Wuhan, China, CL-0137) and BJ human foreskin fibroblasts (ATCC, Manassas, VA, USA, CRL-2522) were maintained in DMEM (Gibco, Thermo Fisher Scientific, Waltham, MA, USA, C11965500BT) supplemented with 10% FBS (Wisent, Saint-Jean-Baptiste, QC, Canada, 085-450) and 1% penicillin–streptomycin at 37 °C, 5% CO_2_, 90–95% humidity (Thermo Forma Series II 3110, Thermo Fisher Scientific, Waltham, MA, USA). Cells at passages 3–5 were used throughout. L929 cells were dissociated with 0.25% trypsin-EDTA; BJ cells with 0.125% trypsin-EDTA.

#### 2.2.2. Matrix Pre-Treatment

Matrix discs (Ø 10 mm) were equilibrated in complete culture medium under standard conditions prior to cell seeding. Pre-degraded matrices were prepared by incubating cell-free discs in complete medium for 7 days, with medium exchanges matching the cell culture schedule, followed by cell seeding for subsequent culture.

#### 2.2.3. Cell Seeding and Experimental Design

For Live/Dead fluorescence and SEM observation, cells were seeded onto matrix discs in 48-well plates (400 µL/well; 2 × 10^4^ cells/well; *n* = 4 per condition; time points: Days 1, 3, 5, 7). For confocal imaging, time points comprised Day 1 and 7 (fresh matrix) and Day 1 and 7 (7-day pre-degraded matrix). For the CCK-8 assay, cells were seeded in 12-well plates (1 mL/well, 2 × 10^4^ cells/well) with matrix discs placed in Transwell inserts to assess indirect-contact cytocompatibility (time points: Days 1, 5); *n* = 3 biological replicates with 4 technical replicates each. Controls consisted of cells cultured on tissue culture polystyrene (TCPS) without matrix.

#### 2.2.4. CCK-8 Viability Assay

Cell viability was quantified using Cell Counting Kit-8 (Solarbio, CA1210, Beijing, China) [35]. At each time point, CCK-8 working solution was added and incubated at 37 °C in the dark for 3 h. Aliquots (100 µL) were transferred to 96-well plates, agitated for 30 s, and absorbance was measured at 450 nm (BioTek Epoch 2, Winooski, VT, USA). Relative viability was calculated as

Relative viability (%) = (*OD_matrix_* − *OD_blank_*)/(*OD_control_* − *OD_blank_*) × 100%

The 70% threshold defined by ISO 10993-5 was adopted as the acceptance criterion for non-cytotoxicity [36]. Each cell line was independently normalized to its own same-day TCPS control.

#### 2.2.5. Live/Dead Fluorescence Staining and Imaging

Cells were washed twice with PBS and stained with calcein-AM/PI (Solarbio, Beijing, China, CA1630; 150 µL/well, 37 °C, 25 min, dark). After one PBS rinse, matrices were inverted onto 20 µL medium to eliminate air bubbles. Widefield fluorescence images were acquired on a Leica Mateo FL microscope (Leica Microsystems, Wetzlar, Germany) (10×/20×; green: Ex 470/40, Em 525/50 nm; red: Ex 546/22, Em 590/33 nm). Confocal imaging was performed on a Zeiss LSM 900 (Carl Zeiss AG, Oberkochen, Germany) (488/561 nm laser lines, 10×/20×) with samples mounted inverted on 0.17 mm glass-bottom dishes. Z-stack imaging was performed where applicable.

#### 2.2.6. SEM Observation of Cell–Matrix Interactions

At designated time points, samples were fixed with 2.5% glutaraldehyde (room temperature, 2 h), dehydrated through a graded ethanol series (10–100%, 15 min per step), critical-point dried (Leica EM CPD300, Leica Microsystems, Wetzlar, Germany), sputter-coated with gold (30 s), and imaged by SEM (Hitachi SU8220, Hitachi High-Tech Corporation, Tokyo, Japan, 5 kV, SE(UL) detector).

#### 2.2.7. Cell Morphological Analysis

Fluorescence images of calcein-AM-stained cells were processed in Fiji/ImageJ (v1.54P) [33,34] using rolling-ball background subtraction, Otsu auto-thresholding, watershed segmentation, and the Analyze Particles function. Shape descriptors extracted per cell included Aspect Ratio (AR = major axis/minor axis of best-fit ellipse), Solidity (cell area/convex hull area), and Elongation Index (EI = (Feret − MinFeret)/(Feret + MinFeret)) [37,38].

L929 cells were further classified into four morphological categories based on combined AR and Solidity thresholds informed by Gaussian Mixture Model analysis of control distributions and established fibroblast morphological descriptions [37]: Round (AR < 1.5 and Solidity ≥ 0.90), Intermediate (1.5 ≤ AR < 2.0 or 0.75 ≤ Solidity < 0.90), Spindle (AR ≥ 2.0 and Solidity ≥ 0.75), and Stellate (Solidity < 0.75). This classification served as a semi-quantitative descriptive tool to visualize overall shifts in morphological distribution rather than a definitive phenotypic assignment [39]. For BJ human fibroblasts, continuous Solidity and EI distributions were compared directly between groups owing to the inherently stellate baseline morphology of primary fibroblasts.

### 2.3. In Vivo Full-Thickness Wound Healing Study

#### 2.3.1. Animals and Grouping

Forty SPF-grade male Sprague–Dawley rats (200–220 g) were obtained from Shanghai SLAC Laboratory Animal Co., Ltd. (Shanghai, China; License No. SCXK(Hu)2022-0004). Animals were individually housed under controlled conditions (20–25 °C, 40–70% RH) with ad libitum access to irradiated maintenance diet and sterile purified water. After a 7-day acclimatization period, rats were randomly allocated into the Fibrous Matrix group (*n* = 20) and the Control group (Vaseline gauze, *n* = 20). Five animals per group were scheduled for sacrifice at Days 7, 14, 21, and 28. On the day of surgery (Day 0), intact skin excised during wound creation in five randomly selected rats was retained as baseline tissue; blood samples were collected simultaneously to serve as references for biochemical and inflammatory marker analyses. All protocols were approved by the IACUC of Hangzhou Hibei Technology Co., Ltd. (Approval No. HB-IACUC-2026011303017KRTK-A; License No. SYXK(Zhe)2023-0020) and conducted in accordance with GB/T 16886.2-2011 [40].

#### 2.3.2. Wound Creation and Intervention

Following a 24 h fast, rats were anesthetized with sodium pentobarbital (40 mg/kg, i.p.). The dorsal skin was shaved and chemically depilated. A full-thickness excisional wound (Ø 20 mm) was created using a biopsy punch, and a silicone splint (inner Ø 25 mm, outer Ø 40 mm, thickness 3 mm) was sutured concentrically to limit wound contraction [41,42,43]. In the Fibrous Matrix group, the composite matrix was applied directly onto the wound bed and left undisturbed throughout the study; a secondary non-adherent gauze covering was placed over the matrix. In the Control group, Vaseline gauze was applied directly onto the wound bed and renewed at each dressing change. All wounds were secured with 3M™ elastic bandage (3M, St. Paul, MN, USA). Dressing changes (secondary gauze in the Fibrous Matrix group; Vaseline gauze in the Control group) were performed twice weekly during the first week (when exudate production was greatest) and once weekly thereafter; wound photography was performed at each change. After Day 14—when the matrix was macroscopically undetectable and wounds no longer required occlusive coverage—dressing changes were discontinued in both groups. This protocol timing was applied identically to both groups.

#### 2.3.3. Wound-Area Reduction and Absorption Assessment

Standardized digital photographs were obtained at Days 0, 3, 7, 14, 21, and 28 with a co-planar ruler for calibration. Wound areas were quantified using ImageJ (NIH, USA). Initial wound area (*A*_0_) was defined as the planimetric area measured immediately after wound creation on Day 0, prior to matrix application. Wound-area reduction was calculated as [44]

Wound-area reduction (%) = (*A*_0_ − *A_t_*)/*A*_0_ × 100%

where *A_t_* denotes residual open wound area at time point *t*.

Macroscopic matrix absorption was scored on a five-point scale: Grade 1 = matrix intact, no visible change; Grade 2 = volume reduction with recognizable shape; Grade 3 = partial fragmentation, original shape partially lost; Grade 4 = trace residue only, integrated with wound tissue; Grade 5 = no macroscopically identifiable matrix material. Body weight was recorded concurrently.

#### 2.3.4. Sample Collection

At each sacrifice time point, rats were euthanized by pentobarbital overdose. Blood was collected, allowed to clot at room temperature for 2 h, and centrifuged (4000 rpm, 10 min) to obtain serum, which was aliquoted and stored at −80 °C. Full-thickness wound-site tissue was excised; one portion was snap-frozen for homogenization and the other fixed in 4% paraformaldehyde for histology.

#### 2.3.5. Serum Oxidative Stress Markers

Serum malondialdehyde (MDA) content was determined by the thiobarbituric acid method (Cat. No. A003-1, Nanjing Jiancheng Bioengineering Institute, Nanjing, China) [45]: 0.1 mL serum was mixed with reagents, heated at 95 °C for 40 min, cooled, centrifuged (4000 rpm, 10 min); supernatant absorbance was read at 532 nm. Serum superoxide dismutase (SOD) activity was measured by the water-soluble tetrazolium salt-1 (WST-1) method (Cat. No. A001-3, Nanjing Jiancheng, Nanjing, China) [46]: 20 µL samples were incubated with enzyme working solution and substrate at 37 °C for 20 min, and absorbance recorded at 450 nm. Both assays were performed on a SpectraMax Plus 384 microplate reader (Molecular Devices, San Jose, CA, USA) following manufacturer protocols.

#### 2.3.6. Tissue Inflammatory Cytokines

Interleukin-6 (IL-6) and interleukin-10 (IL-10) concentrations in wound tissue homogenates were quantified by sandwich ELISA (Cat. No. ml064292 and ml037371, respectively; Mlbio, Shanghai, China). Samples (100 µL) were incubated at 37 °C for 90 min, followed by sequential incubation with biotinylated antibody (1:100, 37 °C, 60 min) and enzyme conjugate (1:100, 37 °C, 30 min), with three and five washing cycles (300 µL wash buffer per well), respectively. Chromogenic substrate was developed at 37 °C for 15 min in the dark before termination with stop solution. Absorbance was read at 450 nm. Results were expressed as pg/mg protein.

#### 2.3.7. Histopathological Evaluation

Paraformaldehyde-fixed specimens were paraffin-embedded and sectioned. H&E staining evaluated inflammatory infiltration, neovascularization, and granulation tissue formation. Masson’s trichrome staining assessed collagen deposition and organization. CD31 immunohistochemistry (IHC) quantified neovascularization. Vimentin/α-smooth muscle actin (α-SMA) immunofluorescence (IF) co-staining characterized fibroblast-to-myofibroblast transition dynamics.

CD31 IHC. Paraffin sections (4 µm) were deparaffinized, rehydrated, and subjected to antigen retrieval in citrate buffer (pH 6.0, 95 °C, 20 min). Endogenous peroxidase was quenched with 3% H_2_O_2_ (10 min). Sections were blocked with 5% normal goat serum (30 min, room temperature) and incubated with rabbit anti-rat CD31 primary antibody (1:200) overnight at 4 °C. HRP-conjugated goat anti-rabbit secondary antibody (1:500) was applied for 50 min at room temperature. DAB chromogen was used for visualization; sections were counterstained with hematoxylin. Vessel density was quantified as CD31^+^ luminal structures per mm^2^ in three randomly selected 200× fields per section by a blinded observer.

Vimentin/α-SMA IF. Frozen sections (8 µm) were fixed in 4% paraformaldehyde (15 min), permeabilized with 0.3% Triton X-100 (10 min), and blocked with 5% BSA (1 h, room temperature). Sections were incubated with mouse anti-Vimentin (1:200) and rabbit anti-α-SMA (1:200) overnight at 4 °C. Secondary antibodies comprised Alexa Fluor 488-conjugated goat anti-mouse (1:500) and Cy3-conjugated goat anti-rabbit (1:500), applied for 1 h at room temperature. Nuclei were counterstained with DAPI. Images were acquired at 200× magnification. The α-SMA^+^/Vimentin^+^ activation ratio was calculated as the mean fluorescence intensity of α-SMA divided by the mean fluorescence intensity of Vimentin across five randomly selected fields per section, analyzed by a blinded observer using ImageJ.

### 2.4. Statistical Analysis

All quantitative data are expressed as mean ± SD. For normally distributed continuous variables (wound-area reduction, body weight, ELISA cytokine concentrations, SOD activity, MDA levels), unpaired Student’s *t*-test was applied for between-group comparisons. For non-normally distributed or bounded continuous data (pore metrics, cell morphology descriptors), the Mann–Whitney *U* test was used. Categorical data (L929 morphological category proportions) were analyzed by a *χ*^2^ test of independence. Semi-quantitative histological inflammation scoring per ISO 10993-6 was compared using unpaired Student’s *t*-test given the sample size (*n* = 5) and additive composite score structure. Comparisons between the Fibrous Matrix and Control groups were performed independently at each time point (*n* = 5 per group per time point); comparisons against the intact skin reference were analyzed separately. CCK-8 viability data comprised *n* = 3 independent biological replicates (each with 4 technical replicates), compared by unpaired Student’s *t*-test. Significance levels: * *p* < 0.05, ** *p* < 0.01, *** *p* < 0.001, **** *p* < 0.0001. In vitro morphological analyses were performed using Python 3.11 (SciPy 1.11); in vivo analyses using GraphPad Prism 9 (GraphPad Software, San Diego, CA, USA). Image analysis was conducted with Fiji/ImageJ (v1.54P) and Image-Pro Plus 6.0 (Media Cybernetics, Rockville, MD, USA). Collagen area fraction was quantified from Masson’s trichrome-stained sections using ImageJ (NIH, USA). Three representative fields per section were captured at 200× magnification. Blue-staining collagen area was segmented using color deconvolution [47] and thresholding, and expressed as a percentage of total tissue area within the wound bed. Three sections per animal were analyzed and averaged to yield a single value per animal (*n* = 5 per group per time point). Collagen area fraction comparisons were performed using Welch’s *t*-test to account for unequal variances between groups.

## 3. Results

### 3.1. Physical Characterization and Structural Evolution of the Fibrous Matrix

The composite fibrous matrix integrates three polymer components—P188 for immediate wettability, PLGA for rapid pore enlargement, and PDO for sustained structural coherence—within a single co-electrospun network. The physicochemical and biological consequences of this staged evolution are characterized below.

**Figure 1:**
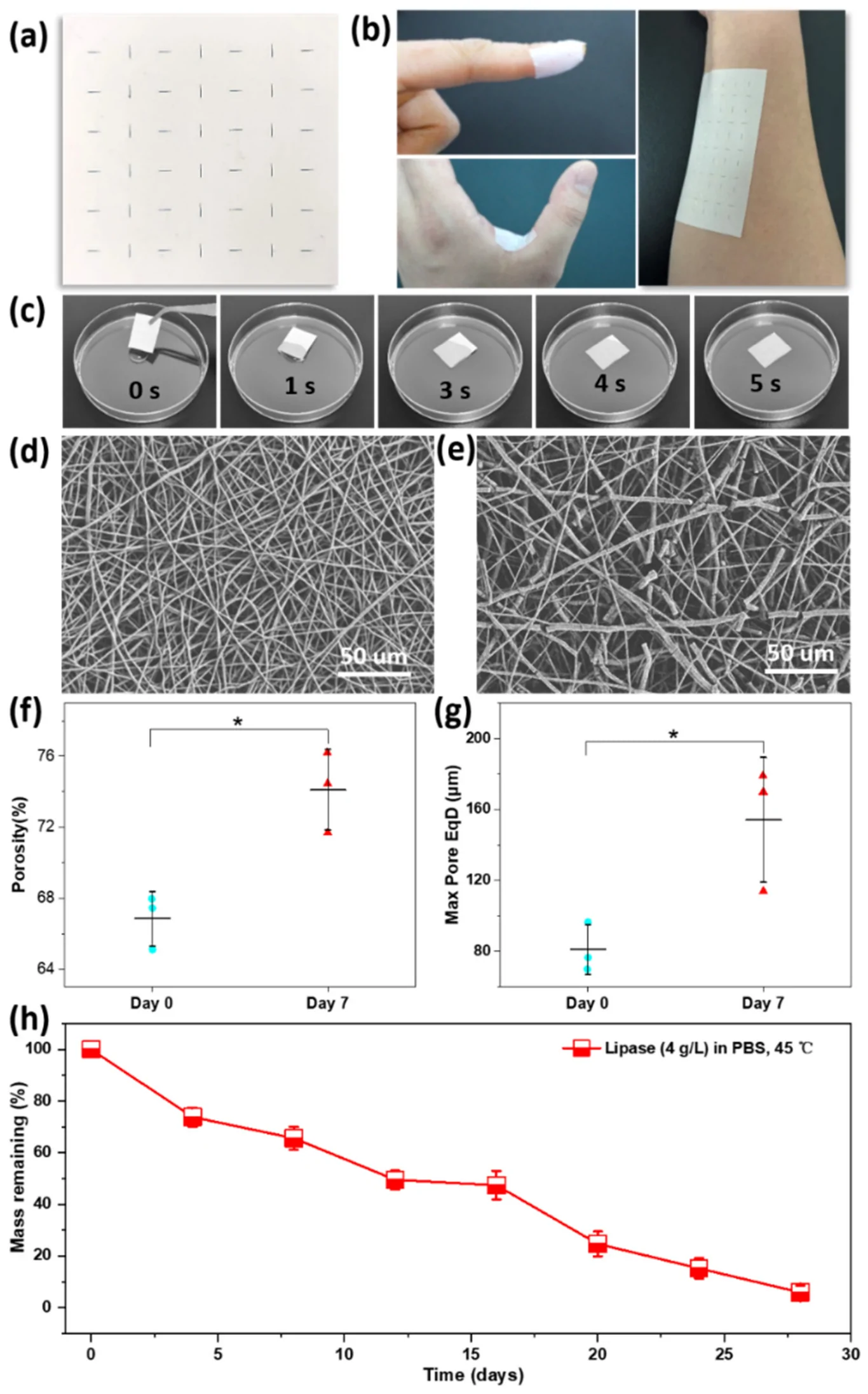
Physical characterization and hydrolysis-associated structural evolution of the composite fibrous matrix. (**a**) Macroscopic appearance of the as-fabricated matrix with uniformly distributed micro-perforations. (**b**) Conformability to curved skin surfaces. (**c**) Wetting kinetics: opaque-to-translucent transition within 5 s of PBS contact. (**d**,**e**) Representative SEM micrographs at Day 0 (**d**) and Day 7 (**e**) in complete culture medium (37 °C, 5% CO_2_). Scale bars = 50 µm. (**f**) Porosity at Day 0 versus Day 7 (*n* = 3; * *p* < 0.05, Mann–Whitney *U* test). (**g**) Maximum pore-equivalent diameter at Day 0 versus Day 7 (*n* = 3; * *p* < 0.05). Data in (**f**,**g**): colored dots represent individual data points; horizontal bars indicate mean; error bars indicate ± SD; image analysis via custom Fiji/ImageJ macro [33,34]. (**h**) Mass remaining (%) under accelerated enzymatic degradation (lipase 4 g/L, PBS, 45 °C; *n* = 3).

Macroscopically, the as-fabricated matrix presents as a flexible, opaque white fibrous sheet characterized by uniformly distributed micro-perforations that facilitate gas exchange and exudate transport (Figure 1a). Upon hydration, the matrix conforms intimately to contoured anatomical surfaces without structural fracture, edge lifting, or the requirement for external fixation (Figure 1b), thereby demonstrating favorable handling characteristics for clinical deployment. This conformability is attributed to hydration-mediated plasticization: P188 dissolution upon aqueous contact establishes hydrophilic channels throughout the fiber network, reducing inter-fiber friction and maximizing network pliability.

Wettability characterization confirmed that the matrix transitions from opaque to translucent within 5 s of aqueous contact (Figure 1c), indicating complete fluid saturation of the fibrous interstices. Drop absorption testing further demonstrated that deposited droplets (3–5 µL) were absorbed instantaneously upon surface contact, with no residual sessile drop observable (Video S1). Contact angle measurement confirmed complete droplet absorption within ~46 ms (effective *θ* = 0°; Figure S2), classifying the matrix as superhydrophilic. Fluid absorption capacity exceeded 300% after 30 min immersion in simulated wound exudate at 37 °C. The matrix exhibited a mean peak tensile load of 13.4 ± 1.4 N (tensile strength ~1.3 MPa) across three independent production lots, with no significant inter-lot variation (*p* > 0.05). This value falls within the reported mechanical range for wound dressings (1–32 MPa) [48] and is consistent with multi-layered commercial wound dressings (0.17–4.60 MPa) [49]. In the absence of P188, electrospun PLGA/PDO fiber mats [12,13] exhibit water contact angles exceeding 110°; P188 incorporation circumvents this hydrophobic barrier by establishing hydrophilic capillary channels upon dissolution, permitting immediate wound-bed application without pre-conditioning.

Scanning electron microscopy (SEM) at Day 0 revealed a dense, randomly oriented fiber network with intact inter-fiber junctions and small, uniform pore spaces (Figure 1d). After 7 days of acellular incubation in complete culture medium (DMEM, 10% FBS, 37 °C, 5% CO_2_), distinct structural remodeling of the scaffold—characterized by segmental fiber fragmentation, localized network discontinuities, and significantly enlarged inter-fiber voids—was evident (Figure 1e). Intermediate and extended time points (Days 5 and 14) confirmed progressive fiber fragmentation and pore enlargement over the incubation period (Figure S3). Quantitative image analysis revealed that porosity increased from 66.8 ± 1.5% to 74.1 ± 2.3% (*p* < 0.05; Figure 1f), and maximum pore-equivalent diameter expanded from 80.4 ± 14.0 µm to 153.6 ± 27.2 µm—a 91% enlargement (*p* < 0.05, Mann–Whitney *U* test, *n* = 3; Figure 1g). This Day 7 pore dimension falls within the range reported to support active dermal fibroblast infiltration in electrospun scaffolds [16,17], consistent with staged hydrolysis yielding a biologically relevant pore architecture without post-fabrication modification.

Bulk degradation kinetics were further characterized under accelerated enzymatic conditions (lipase 4 g/L, PBS, 45 °C; Figure 1h). Mass remaining declined progressively from 100% at Day 0 to 73.8 ± 3.7% by Day 4 and 65.6 ± 4.4% by Day 8, consistent with early hydrolytic attack on PLGA fiber junctions concurrent with residual P188 dissolution. By Day 12, approximately half of the initial mass had been consumed (49.5 ± 3.6%), followed by a transient plateau through Day 16 (47.4 ± 5.5%), indicative of a transition from preferential surface erosion to bulk fragmentation of the remaining PDO backbone. Rapid mass loss resumed thereafter, with only 24.7 ± 4.8% remaining at Day 20 and 5.9 ± 2.6% at Day 28. This degradation profile indicates near-complete mass loss within 28 days under enzymatic challenge—temporally consistent with the matrix being macroscopically undetectable in vivo by Day 14 (Grade 5; Section 3.3.1), where cellular enzymatic activity, mechanical loading, and physiological temperature collectively accelerate matrix consumption beyond in vitro rates.

### 3.2. Fibroblast Behavior on the Fibrous Matrix

#### 3.2.1. In Vitro Cytocompatibility and Fibroblast Morphological Response

The functional implications of progressive structural evolution were assessed using two fibroblast models of complementary relevance: L929 murine fibroblasts, the reference cell line specified by ISO 10993-5 for standardized cytotoxicity evaluation [36], and BJ neonatal human foreskin fibroblasts, which more closely approximate the proliferative and migratory phenotype of dermal fibroblasts in regenerating wound tissue.

**Figure 2:**
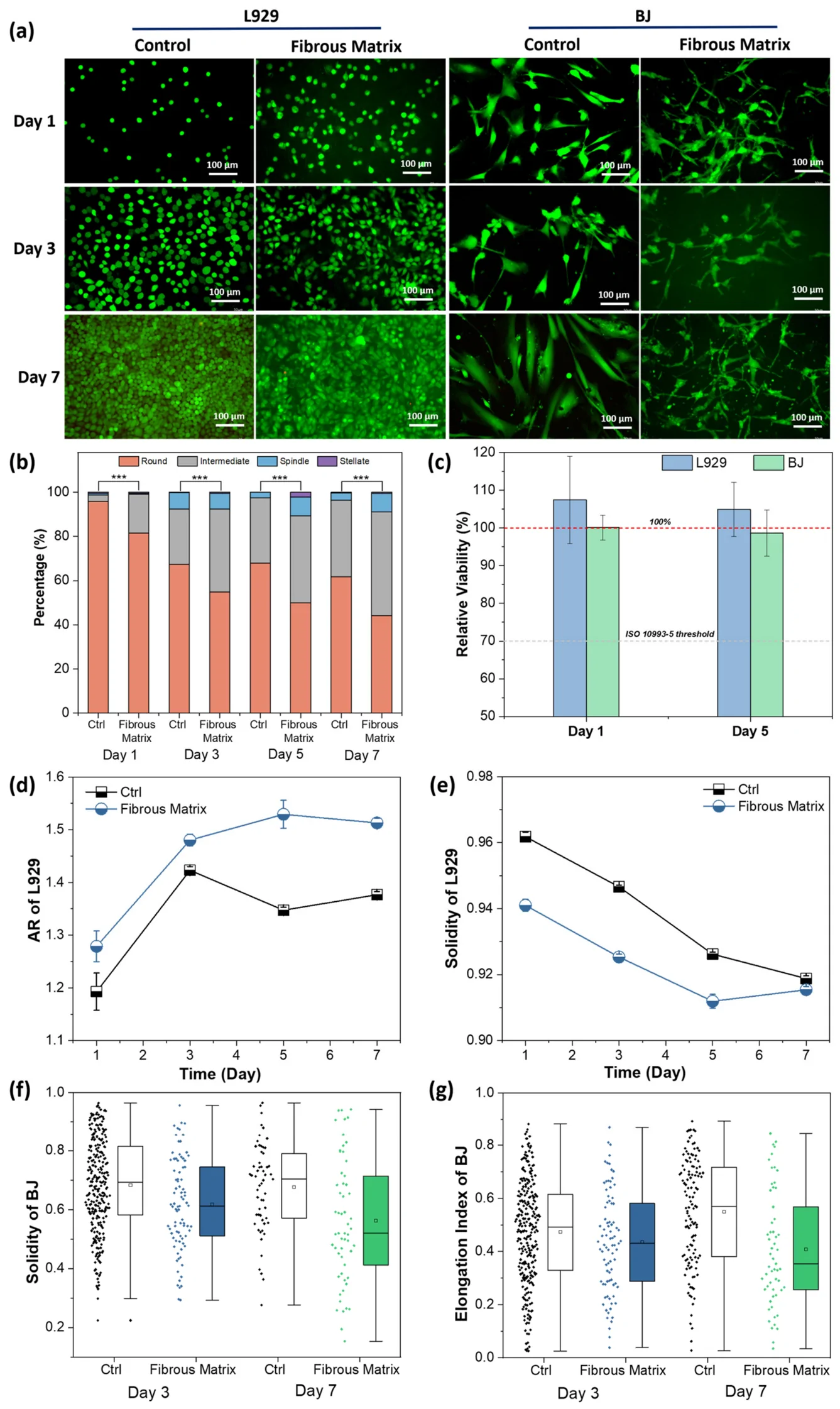
In vitro cytocompatibility and morphometric characterization of fibroblasts cultured on the composite fibrous matrix. (**a**) Representative calcein-AM fluorescence images of L929 and BJ fibroblasts on TCPS (Control) and composite matrix at Days 1, 3, and 7. Scale bars = 100 µm. (**b**) Discrete morphological classification of L929 cells into Round, Intermediate, Spindle, and Stellate categories (*** *p* < 0.001, *χ*^2^ test; *n* ≥ 150 cells per condition). (**c**) Relative metabolic viability (CCK-8) of L929 and BJ fibroblasts at Days 1 and 5 (indirect contact, Transwell). Data: mean ± SD, *n* = 3 biological replicates. Red dashed line = 100% (control); grey dashed line = 70% (ISO 10993-5 threshold). ns, not significant vs. same-day control (unpaired Student’s *t*-test). (**d**,**e**) Aspect ratio (**d**) and Solidity (**e**) of L929 cells over 7 days (mean ± SD; *n* ≥ 50 cells per time point). (**f**,**g**) Solidity (**f**) and Elongation Index (**g**) of BJ fibroblasts at Days 3 and 7; box plots show median, interquartile range, and individual data points.

Morphological observation. Fluorescence imaging of calcein-AM-labeled cells documented progressive, substrate-dependent divergence in fibroblast morphology over the 7-day culture period (Figure 2a). At Day 1, cells on both the composite matrix and TCPS control exhibited predominantly spherical morphologies with comparable attachment densities, indicating comparable initial attachment. By Day 3, fibroblasts on the composite matrix had adopted elongated and stellate configurations with discernible lamellipodial extensions, whereas control cells retained compact, polygonal profiles. By Day 7, matrix-cultured cells displayed extensively branched morphologies with multi-directional cytoplasmic projections. This substrate-dependent divergence was observed in both L929 and BJ populations, indicating a conserved morphological response to the evolving fibrous architecture.

Metabolic viability. CCK-8 analysis demonstrated that the composite fibrous matrix did not adversely affect cellular metabolic activity (Figure 2c). Relative viability of L929 cells was 107.4 ± 4.2% at Day 1 and 104.9 ± 3.8% at Day 5; BJ cells yielded 98.6 ± 5.1% and 103.2 ± 4.6% at corresponding time points. All measurements exceeded the 70% non-cytotoxicity threshold defined by ISO 10993-5, indicating that any soluble degradation products—including lactic acid, glycolic acid, and poloxamer fragments—remained at sub-cytotoxic concentrations throughout the culture period. Values marginally exceeding 100% are consistent with reported poloxamer membrane-stabilizing effects at sub-micellar concentrations [50]. No statistically significant differences were detected between matrix and control groups for either cell line at any time point (unpaired Student’s *t*-test, all *p* > 0.05).

Discrete morphological classification (L929). Categorization into four morphological classes revealed progressive redistribution of population composition on the composite matrix (Figure 2b). At Day 1, both groups were dominated by the Round category (>60%); however, the matrix group already exhibited a significantly higher proportion of Intermediate and Spindle phenotypes relative to the control (*χ*^2^ test: *p* < 0.001). From Day 3 onward, matrix-cultured cells exhibited a further shift toward Spindle and Stellate phenotypes with concomitant depletion of the Round fraction (*χ*^2^ test: *p* < 0.001 at Days 3, 5, and 7). This redistribution is temporally congruent with pore enlargement kinetics (Section 3.1), consistent with expanding inter-fiber spaces progressively permitting cytoskeletal extension.

Continuous shape descriptors (L929). Complementary geometric analysis using continuous morphometric parameters provided further resolution (Figure 2d,e). AR was significantly elevated on the composite matrix relative to TCPS at every time point (*p* < 0.001, Mann–Whitney *U* test), indicating sustained cellular elongation. AR on the matrix increased from ~1.27 (Day 1) to a peak of ~1.50 (Day 3) and remained elevated through Day 7 (~1.46), whereas the control group showed a more gradual rise. Simultaneously, Solidity on the composite matrix decreased from ~0.96 (Day 1) to a minimum of ~0.91 (Day 5) before stabilizing at ~0.92 (Day 7), indicating increasing contour irregularity attributable to peripheral protrusion formation. The concurrent AR elevation and Solidity reduction constitute a morphometric signature distinguishing multi-directional spreading from simple uniaxial elongation: the latter would increase AR while maintaining high Solidity, whereas the former generates irregular, high-perimeter outlines characteristic of fibroblasts extending exploratory lamellipodia across topographically heterogeneous substrates [37,38].

Cross-species validation (BJ human fibroblasts). Cross-species applicability was assessed through continuous distributional analysis of BJ human dermal fibroblast morphology (Figure 2f,g). At Day 3, BJ cells on the composite matrix displayed reduced median Solidity (0.62 vs. 0.68) and reduced median EI (0.44 vs. 0.47) relative to TCPS. By Day 7, this separation was further accentuated (Solidity: 0.56 vs. 0.66; EI: 0.41 vs. 0.55). The concurrent reduction in both metrics is geometrically inconsistent with uniaxial polarization—which would elevate EI—and instead denotes multi-polar cytoplasmic extension. EI trajectories diverged: control cells showed progressive uniaxial polarization (from 0.47 to 0.55) whereas EI of matrix-cultured cells declined (from 0.44 to 0.41), indicating that the evolving fibrous topography counteracts uniaxial elongation and promotes multi-directional spreading. The broader interquartile ranges on the composite matrix reflect morphological heterogeneity arising from locally variable pore dimensions within the stochastic fiber network.

Integrative interpretation. Together, both analytical approaches confirm that the composite fibrous matrix elicits progressive, multi-directional fibroblast spreading temporally aligned with pore enlargement kinetics (Section 3.1).

#### 3.2.2. Multi-Modal Visualization of Cell–Matrix Interactions

To spatially resolve the cell–substrate engagement underlying the morphometric changes documented in Section 3.2.1, confocal fluorescence microscopy and scanning electron microscopy (SEM) were employed to characterize fibroblast–fiber interactions at single-cell resolution.

**Figure 3:**
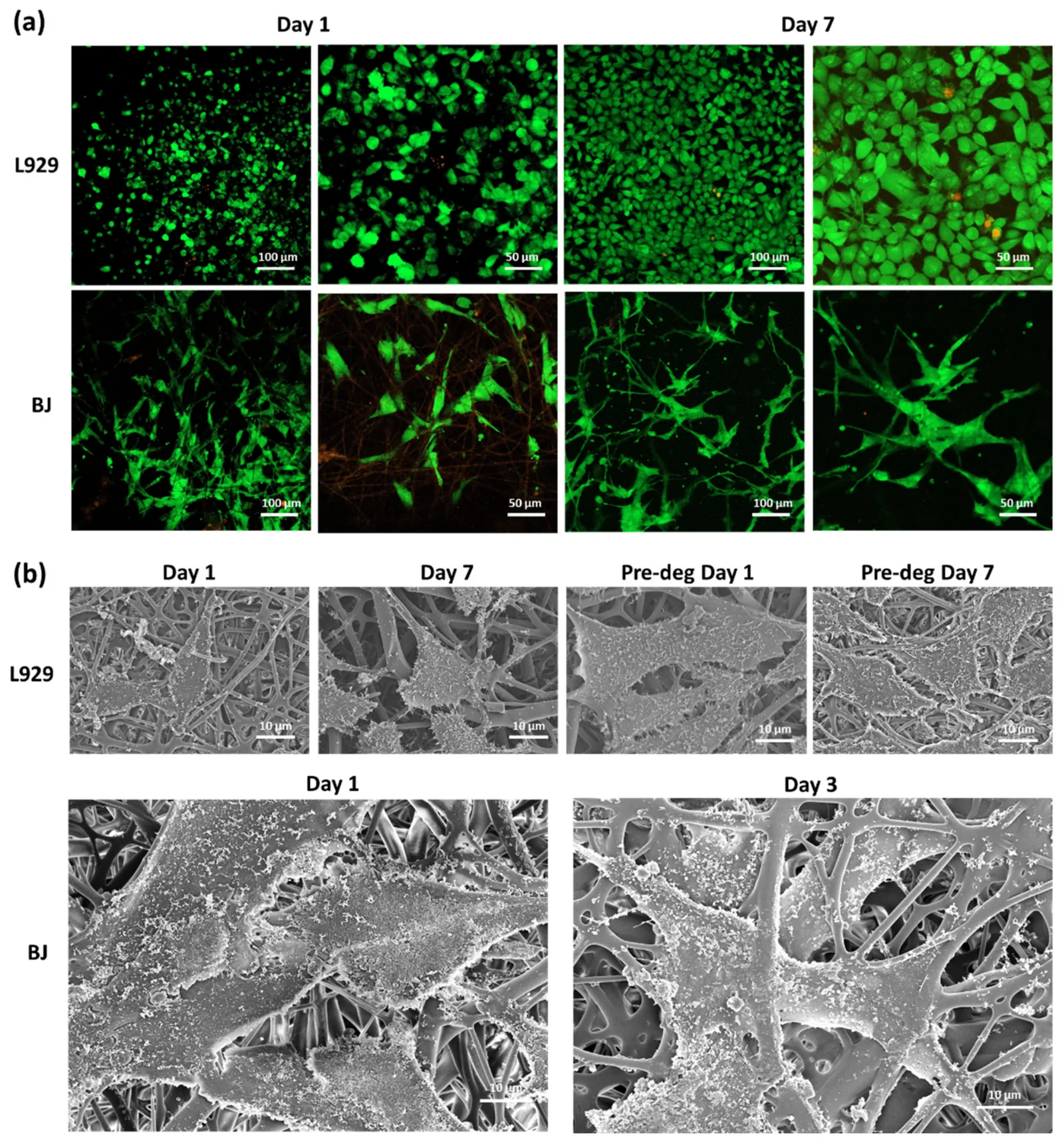
Multi-modal visualization of fibroblast–matrix interactions on the composite fibrous matrix. (**a**) Confocal fluorescence micrographs (calcein-AM/PI; green = viable, red = non-viable) of L929 (upper panels) and BJ (lower panels) fibroblasts at Day 1 and Day 7. Left columns: low magnification (scale bar = 100 µm); right columns: high magnification (scale bar = 50 µm). Red fibrous background in BJ panels represents matrix autofluorescence. (**b**) Scanning electron micrographs of cell–fiber interactions. Upper row: L929 on fresh matrix (Day 1, Day 7) and on 7-day pre-degraded matrix (Pre-deg Day 1, Pre-deg Day 7); scale bars = 10 µm. Lower row: BJ on fresh matrix (Day 1, Day 3); scale bars = 10 µm.

Live/dead fluorescence staining (calcein-AM/PI) of L929 and BJ fibroblasts cultured directly on the composite matrix demonstrated sustained viability throughout the 7-day observation period (Figure 3a). At Day 1, L929 cells presented predominantly spherical-to-ovoid morphologies with limited cytoplasmic extension. By Day 7, these cells had transitioned to flattened, polygonal configurations with well-defined margins, corroborating the increased aspect ratio and Stellate fraction quantified in Figure 2b,d.

BJ fibroblasts exhibited a more rapid morphological transition. Stellate spreading with multi-directional lamellipodial extensions was already prominent at Day 1, progressing to branched architectures with elongated cytoplasmic processes spanning adjacent inter-fiber junctions by Day 7. The earlier spreading onset in BJ cells is consistent with the higher migratory capacity characteristic of primary human dermal fibroblasts.

SEM provided direct visualization of physical cell–matrix interactions at the individual fiber scale (Figure 3b, upper row). On fresh matrix at Day 1, L929 cells appeared as rounded bodies situated atop the fiber network with minimal substrate contact area. By Day 7, cells had flattened against the fibrous substrate and extended thin filopodial projections along adjacent fibers, establishing multi-point adhesion across an expanded contact interface. BJ fibroblasts cultured on fresh matrix displayed extensive lamellipodial sheets bridging inter-fiber voids by Day 3 (Figure 3b, lower row), with cytoplasmic processes enveloping individual fibers—ultrastructural morphology consistent with the reduced Solidity values reported in Figure 2f.

To delineate the respective contributions of matrix architectural state and culture duration to the observed spreading response, L929 cells were seeded onto composite matrices that had been pre-degraded for 7 days in cell-free complete medium prior to cell introduction (Figure 3b, Pre-deg panels). At Day 1 post-seeding, cells on pre-degraded matrices already exhibited spreading characteristics—flattened cell bodies, extended cytoplasmic processes, and multi-point fiber contact—comparable to those observed on fresh matrices only after 7 days of culture. By Day 7 on pre-degraded substrates, spreading had advanced further, with continuous cytoplasmic lamellae spanning multiple pore spaces. This observation is consistent with the enlarged pore architecture resulting from PLGA hydrolysis—rather than culture duration or soluble-factor signaling—constituting a primary structural correlate of fibroblast spreading. Quantitative morphometric analysis confirmed that L929 cells at Day 1 on pre-degraded matrices were significantly shifted from Fresh Day 1 toward Fresh Day 3 in all descriptors (all *p* < 0.001), with category distributions directionally closer to Fresh Day 3 than to Fresh Day 1, consistent with accelerated accommodation by approximately 2 days. By Day 7, Pre-deg and Fresh matrices yielded morphologically indistinguishable profiles (all *p* > 0.05; Table S1). The more advanced morphology observed in SEM (Figure 3b) reflects ultrastructural features not captured by fluorescence-based morphometric descriptors. Confocal Z-stack imaging indicated cells present at multiple focal planes within the matrix (Figure S4; Video S2).

### 3.3. Full-Thickness Excisional Wound Healing in a Splinted Dorsal Model

#### 3.3.1. Wound-Area Reduction Kinetics

**Figure 4:**
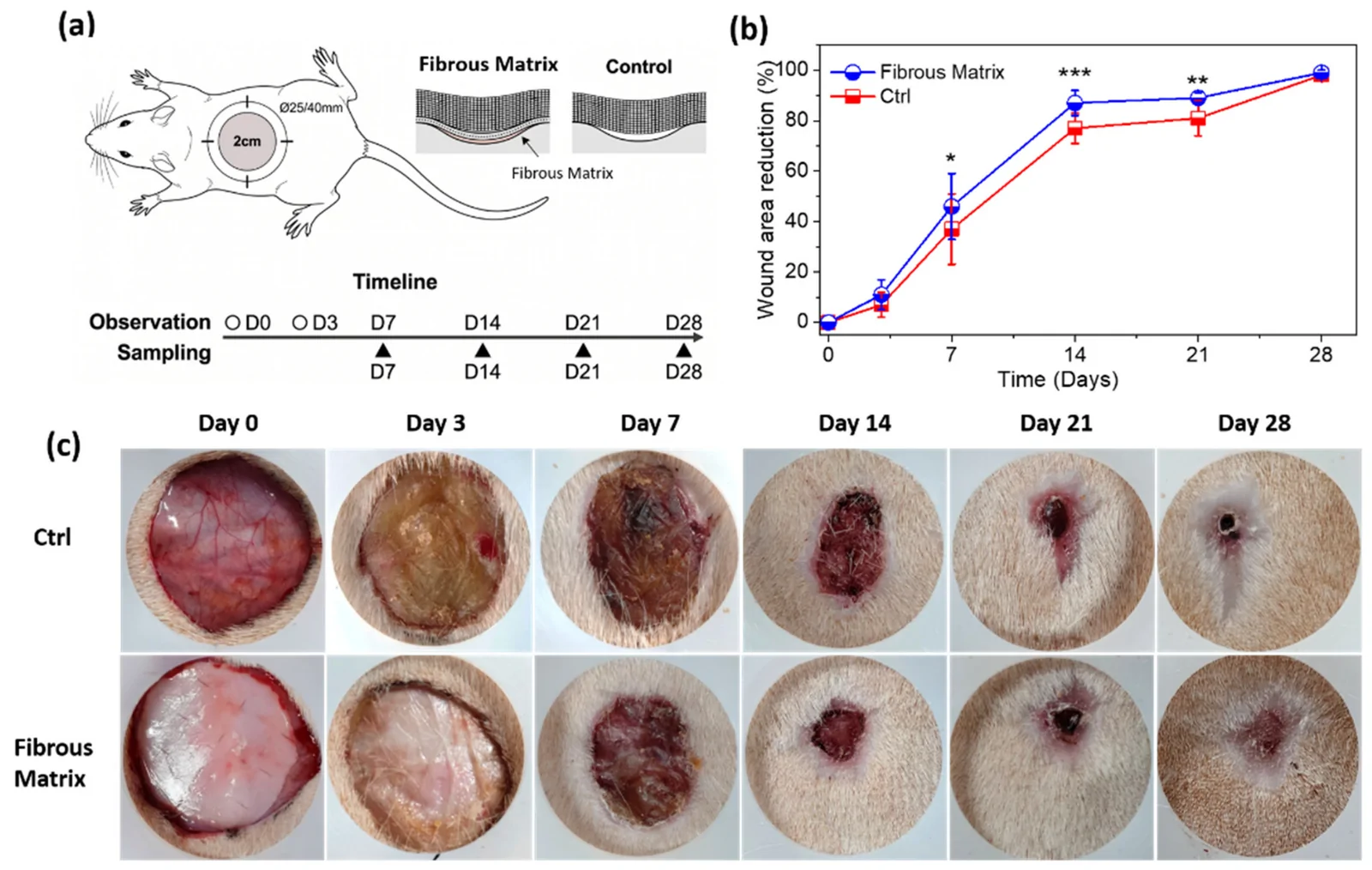
Macroscopic evaluation of wound healing in a splinted full-thickness excisional rat model. (**a**) Schematic of experimental design: full-thickness defects (Ø 20 mm) stabilized with silicone splints; timeline indicates observation (D0–D28) and tissue sampling (D7, D14, D21, D28) time points. (**b**) Wound-area reduction (%) over 28 days (mean ± SD; *n* = 20 at D0–D7, *n* = 15 at D14, *n* = 10 at D21, *n* = 5 at D28 due to sequential sacrifice). * *p* < 0.05, ** *p* < 0.01, *** *p* < 0.001 vs. Control. (**c**) Representative wound photographs at each time point for Control (**upper**) and Fibrous Matrix (**lower**) groups.

The Sprague–Dawley rat was selected for its well-characterized wound healing physiology, extensive literature benchmarks for histological and molecular endpoints, and sufficient body size to accommodate clinically relevant wound dimensions (Ø 20 mm) with reliable surgical reproducibility [43,44]. To minimize wound contraction inherent to loose-skinned rodents and better approximate human-like healing through granulation and re-epithelialization, a silicone splint (inner Ø 25 mm, outer Ø 40 mm, thickness 3 mm) was sutured concentrically around each wound [41,42]. This configuration constrains panniculus carnosus-driven contraction, ensuring that measured wound-area reduction predominantly reflects tissue regeneration rather than mechanical skin retraction (Figure 4a).

Baseline wound areas at Day 0 were comparable between groups (Control: 4.50 ± 0.28 cm^2^; Fibrous Matrix: 4.66 ± 0.30 cm^2^; *p* > 0.05), indicating balanced surgical randomization. Quantitative planimetric analysis revealed that matrix-treated wounds achieved significantly greater area reduction than Vaseline gauze controls during the proliferative phase (Figure 4b). Intergroup separation emerged at Day 7 (Fibrous Matrix: 46 ± 13% vs. Control: 37 ± 14%; *p* < 0.05), reached maximum statistical significance at Day 14 (87 ± 5% vs. 77 ± 6%; *p* < 0.001), and persisted through Day 21 (89 ± 2% vs. 81 ± 7%; *p* < 0.01). By Day 28, both groups converged toward near-complete closure (99 ± 1% vs. 98 ± 2%; *p* > 0.05), consistent with the expected ceiling effect as wounds approach terminal re-epithelialization [51]. The temporal concentration of the therapeutic effect within the Day 7–21 window coincides with the period of active granulation tissue formation and epithelial migration, consistent with the composite matrix contributing principally during the inflammatory and proliferative phases rather than the remodeling phase.

Representative photographs documented gross wound progression (Figure 4c). At Day 3, wound beds in both groups appeared moist and granulating with no discernible intergroup difference. By Day 7, matrix-treated wounds demonstrated visibly advanced wound margins with emerging epithelial fronts, whereas control wounds retained broader exposed granulation surfaces. At Day 14, matrix-treated wounds exhibited well-advanced epithelial coverage with minimal residual eschar, whereas controls retained larger open areas with persistent crusting. By Day 28, both groups achieved near-complete closure with well-integrated neo-tissue.

Macroscopic assessment revealed that matrix absorption proceeded in temporal coordination with wound-area reduction: Grade 2 (volume reduction with recognizable shape) at Day 3; Grade 4 (trace residue integrated within wound tissue) by Day 7; and Grade 5 (no macroscopically identifiable material) from Day 14 onward. This macroscopic grading reflects structural integration into regenerating tissue; complete molecular absorption of the PDO component continues over subsequent months, consistent with its established degradation profile.

#### 3.3.2. Histological Evaluation of Tissue Repair

**Figure 5:**
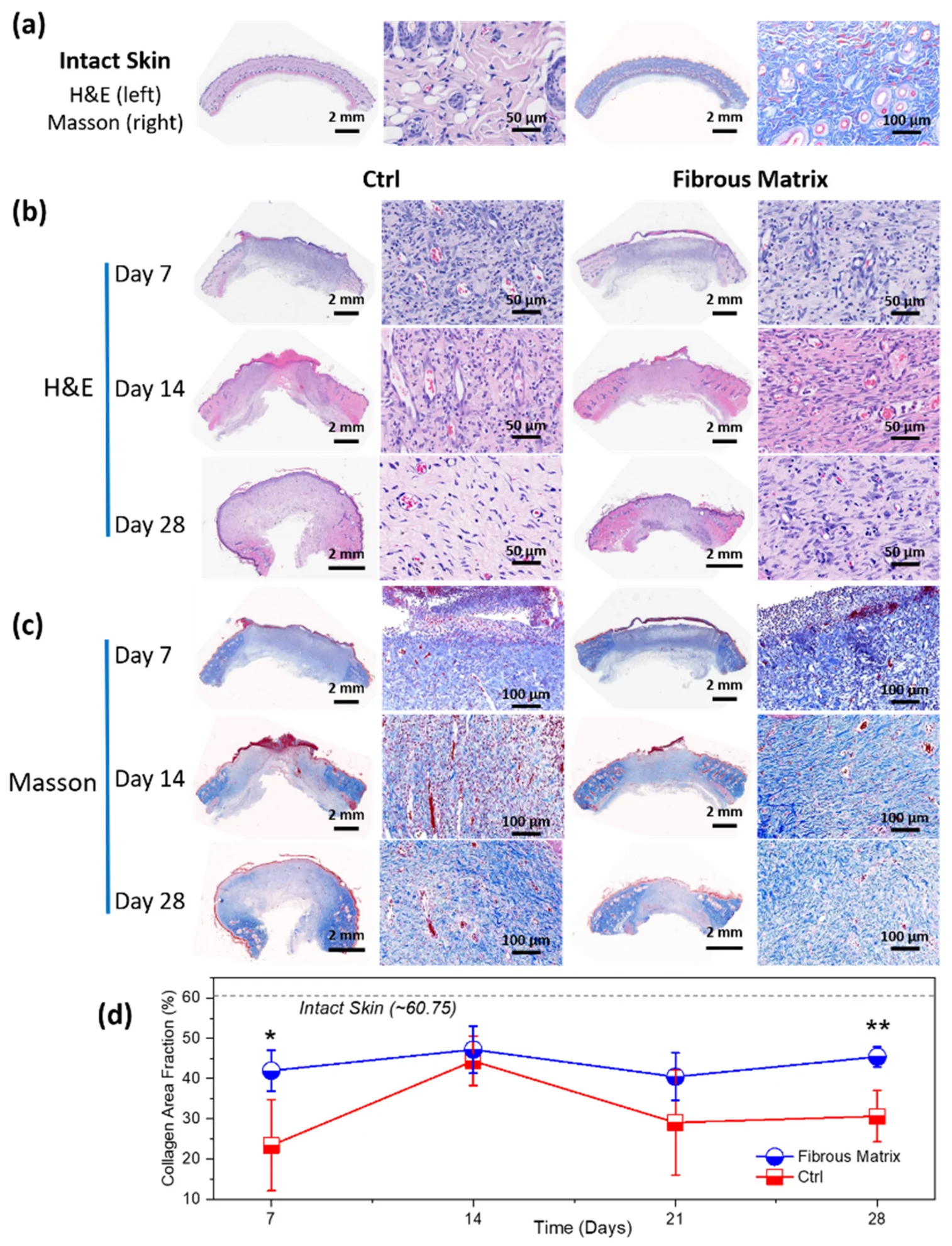
Histological evaluation of wound healing. (**a**) Representative images of intact skin baseline (H&E, left; Masson’s trichrome, right). (**b**) Representative H&E-stained sections at Days 7, 14, and 28. (**c**) Representative Masson’s trichrome-stained sections at corresponding time points. Overview (left): full wound cross-section; magnified insets (right): granulation tissue region. H&E: 400×; Masson’s trichrome: 200×. Scale bars indicated in each panel. Semi-quantitative inflammation scoring detailed in Table 1. (**d**) Quantitative collagen area fraction (%) determined from Masson’s trichrome-stained sections using ImageJ. Data: mean ± SD, *n* = 5 per group per time point; each time point represents independent animal cohorts due to sequential sacrifice design. Dashed line indicates intact skin baseline (~60.75%). Statistical comparison: Welch’s *t*-test at each time point; * *p* < 0.05, ** *p* < 0.01. Lines connecting data points are shown to visualize temporal trends.

Histological assessment was performed using H&E and Masson’s trichrome staining at Days 7, 14, and 28, with intact skin serving as the morphological reference (Figure 5a–c).

Inflammatory response and biocompatibility. Semi-quantitative scoring per ISO 10993-6:2016 at 400× magnification (Table 1) revealed a significantly lower composite inflammation score in the Fibrous Matrix group at Day 7—the only time point at which residual matrix material was histologically identifiable—relative to controls (10.27 ± 1.28 vs. 12.07 ± 1.49; *p* = 0.0014), primarily reflecting a decreased polymorphonuclear cell density in the peri-wound zone. High-magnification cross-sectional imaging at Day 7 confirmed fibroblast-like cells distributed among residual scaffold fibers, with neutrophils present in the peri-fiber zone (Figure S5). From Day 14 onward, scores declined progressively in both groups and were statistically indistinguishable (*p* = 0.52, 0.42, and 0.64 at Days 14, 21, and 28, respectively), indicating convergent remodeling trajectories following complete matrix absorption. Scores in both groups remained elevated relative to intact skin (1.47 ± 1.51; *p* < 0.0001 at all time points), consistent with ongoing tissue remodeling through Day 28. No foreign body giant cells or fibrous encapsulation were identified in any specimen; only minimal focal necrosis was observed in isolated sections at Day 7 in both groups, resolving completely by Day 14. Overall, these findings—absence of foreign body giant cells, absence of fibrous encapsulation, and progressive inflammation resolution indistinguishable from the clinical control from Day 14 onward—are consistent with favorable biocompatibility as assessed within the ISO 10993-6 semi-quantitative framework.

Tissue architecture and collagen deposition. Representative H&E and Masson’s trichrome staining revealed accelerated early tissue organization in the Fibrous Matrix group during matrix residence, followed by intergroup convergence after absorption (Figure 5b,c). At Day 7, the Fibrous Matrix group displayed a thicker granulation layer with early fibroblast alignment (H&E) and more extensive blue-staining collagen (Masson’s trichrome), whereas controls exhibited predominantly red-staining immature granulation tissue with sparse, disorganized fibrils. Quantitative analysis confirmed significantly greater collagen area fraction in the Fibrous Matrix group (41.9 ± 5.1% vs. 23.4 ± 11.4%; *p* < 0.05; Figure 5d). By Day 14, collagen area fraction converged between groups (47.2 ± 5.9% vs. 44.4 ± 6.3%; *p* > 0.05), reflecting equivalent synthesis at the proliferative peak. Thereafter, the Control group exhibited a pronounced decline (29.0% at Day 21), whereas the Fibrous Matrix group maintained stable collagen levels (range: 40.4–47.2%) [20,52]. The Day 21 intergroup difference did not reach significance (*p* = 0.13) owing to high control-group variability. By Day 28, H&E revealed reduced cellularity with maturing connective tissue in both groups, with denser collagen organization confirmed by Masson’s trichrome in the Fibrous Matrix group (45.4 ± 2.6% vs. 30.6 ± 6.4%; *p* < 0.01; Figure 5d). Neither group had reached intact skin baseline (~60.7%), consistent with the extended remodeling timeline of rodent wound models [20].

This temporal pattern—early accelerated deposition during matrix residence (Day 7), transient convergence at the proliferative peak (Day 14), and a significant intergroup difference re-established at Day 28 (*p* < 0.01)—is consistent with scaffold-associated tissue organization conferring a collagen benefit that persists beyond matrix absorption. The mechanistic basis for this differential retention is examined in Section 4.4.

Neovascularization (CD31). CD31^+^ vessel density was significantly elevated in both groups relative to intact skin at Day 7 (Control: 48.27 ± 13.52; Fibrous Matrix: 44.53 ± 6.81 vs. Normal: 11.07 ± 5.89 vessels/mm^2^; *p* < 0.0001) and declined progressively through Day 28, consistent with physiological vascular maturation. No significant intergroup differences were detected at any time point (all *p* > 0.05), consistent with the observed healing acceleration being associated with structural facilitation of cellular organization rather than through enhanced angiogenesis.

#### 3.3.3. Myofibroblast Activation Dynamics

**Figure 6:**
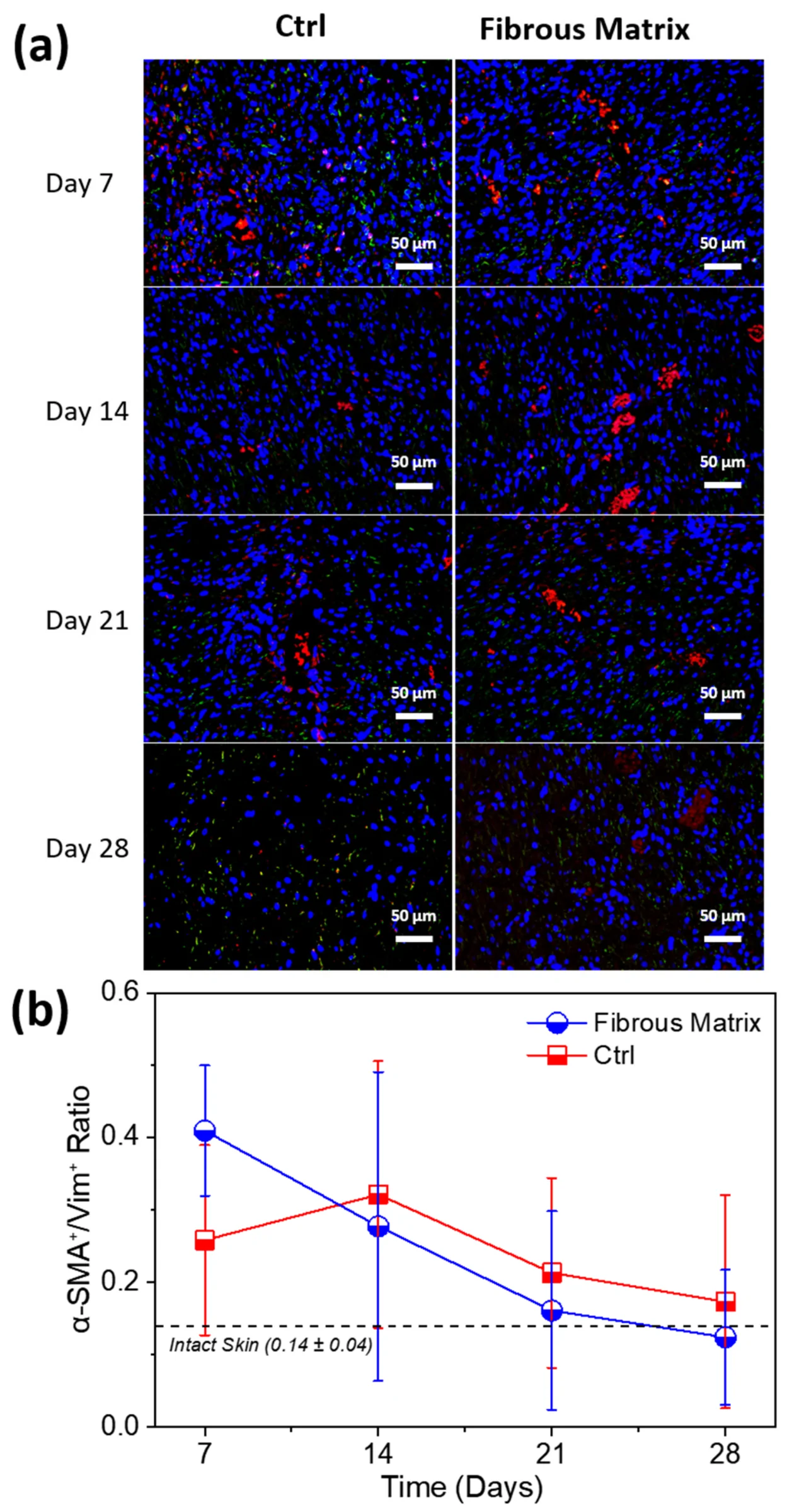
Myofibroblast activation dynamics. (**a**) Representative immunofluorescence at Days 7, 14, 21, and 28 (Vimentin, green; α-SMA, red; nuclei (DAPI), blue). Scale bars = 50 µm. (**b**) Activation ratio over time (mean ± SD, *n* = 5). Dashed line: intact skin baseline (0.14 ± 0.04). *p* > 0.05 at all time points (unpaired *t*-test).

Vimentin/α-SMA dual immunofluorescence was employed to assess myofibroblast activation across the healing timeline. The activation ratio (α-SMA^+^/Vimentin^+^ fluorescence intensity) was quantified at each time point (Figure 6a,b).

The Fibrous Matrix group exhibited a biphasic activation pattern. At Day 7, the activation ratio was elevated relative to controls (0.41 ± 0.09 vs. 0.26 ± 0.13), consistent with the fibrous microstructure of the matrix providing mechanical cues that promote contractile phenotype acquisition [53,54]. From Day 14 onward, the activation ratio in the Fibrous Matrix group declined below that of controls (Day 14: 0.28 ± 0.21 vs. 0.32 ± 0.18; Day 21: 0.16 ± 0.14 vs. 0.21 ± 0.13; Day 28: 0.12 ± 0.09 vs. 0.17 ± 0.15), approaching the intact skin baseline more rapidly.

Although individual time-point comparisons did not reach statistical significance (*p* > 0.05; *n* = 5), the biphasic trajectory—elevated activation during matrix residence followed by accelerated resolution—was consistently observed [21,54].

#### 3.3.4. Systemic Safety and Oxidative Stress Profile

All animals remained in good general health throughout the observation period, with no adverse events, wound infections, or signs of systemic toxicity. Body weight increased comparably in both groups from approximately 209 g to 298 g over 28 days (Table 2; all *p* > 0.05), indicating the absence of gross systemic toxicity attributable to the matrix.

Tissue IL-6 and IL-10 in wound homogenates were elevated in both groups relative to intact skin (*p* < 0.05 at most time points) but showed no intergroup differences (all *p* > 0.05), indicating that the composite matrix neither amplified nor suppressed local cytokine responses. Serum oxidative stress markers diverged only at Day 28: SOD activity was higher in the Fibrous Matrix group (21.70 ± 0.52 vs. 20.82 ± 0.28 U/mL; *p* = 0.010), while MDA was lower (7.48 ± 2.75 vs. 13.07 ± 3.26 nmol/mL; *p* = 0.019); no intergroup differences were detected at Days 7–21 (all *p* > 0.05). These late-phase divergences suggest that accelerated wound resolution may be associated with reduced cumulative oxidative burden [55], although causality remains to be established.

## 4. Discussion

This study aimed to determine whether a fully synthetic absorbable composite electrospun matrix—comprising three polymer components integrated within a single fiber network to contribute distinct yet complementary functions—can serve as an effective dermal scaffold for full-thickness wound repair. Specifically, we examined whether a staged structural remodeling process, associated with progressive hydrolytic degradation, could convert an initially dense electrospun architecture into a dynamically evolving scaffold. We hypothesized that this temporal evolution would provide a pore architecture supporting progressive fibroblast accommodation, would structurally adapt to meet the changing demands of the entire healing process, and would achieve macroscopic absorption without adverse tissue reactions. The physicochemical, in vitro, and in vivo data presented here collectively support this hypothesis: the Fibrous Matrix satisfies these competing biological demands through a single fabrication step, translating its uniquely evolving microenvironment into measurable early cellular accommodation, accelerated wound repair, and durable collagen architecture in a preclinical model.

### 4.1. Resolving the Porosity–Integrity Dilemma Through Temporal Component Partitioning

**Scheme 1:**
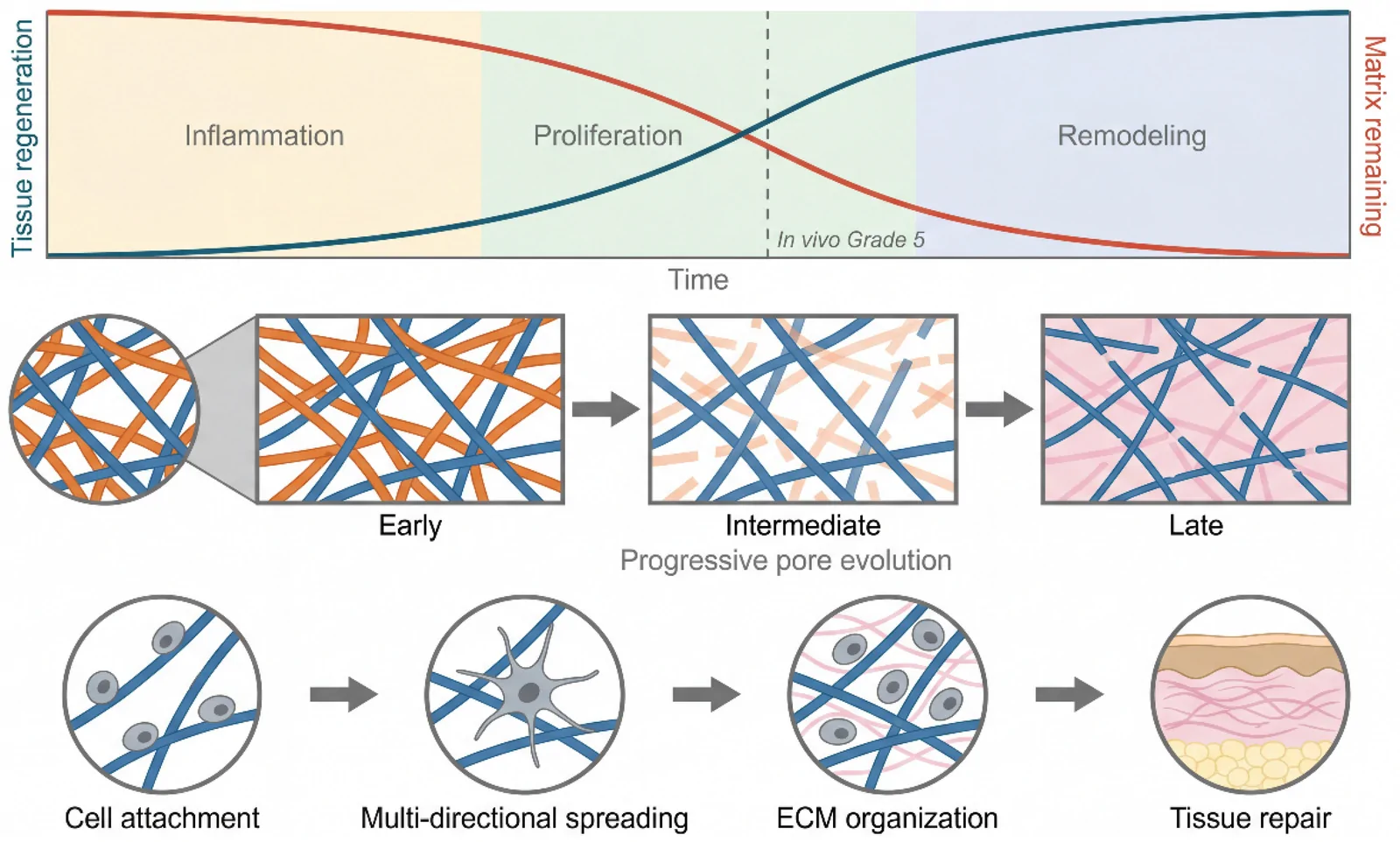
Staged absorption of the composite fibrous matrix and its functional consequences. Top: Conceptual temporal relationship between tissue repair (teal curve) and matrix mass remaining (coral curve) over 28 days; dashed vertical line indicates macroscopically undetectable in vivo (Grade 5, Day 14). The x-axis spans the inflammatory (I), proliferative (P), and remodeling (R) phases. Middle: Progressive pore adaptation as the rapidly absorbing PLGA component (orange fibers) is consumed while the slow-absorbing PDO backbone (blue fibers) maintains structural continuity; pink fill denotes tissue ingrowth. Pore-equivalent diameter values (Day 0: ~80 µm; Day 7: ~154 µm) are indicated. Bottom: Sequential biological responses on the evolving matrix—initial wettability (P188, seconds), fibroblast accommodation and multi-directional spreading (PLGA hydrolysis, days), and sustained mechanical support during tissue maturation (PDO, weeks–months). Quantitative data supporting each stage are presented in Figure 1, Figure 2, Figure 3, Figure 4, Figure 5 and Figure 6.

A central constraint of conventional electrospun scaffolds is that the dense fiber packing conferring mechanical continuity simultaneously restricts inter-fiber pore dimensions below the range required for three-dimensional fibroblast infiltration [9,14]. As noted in the Introduction, single-polymer pore-enlargement strategies yield static architectures that cannot adapt to evolving biological demands [10,18,56]. The composite design described herein addresses this constraint by assigning each functional demand to a dedicated polymer component absorbed at a rate matched to its biological role (Scheme 1). P188 provides immediate wettability and hydration-mediated conformability within 5 s (Figure 1c); PLGA hydrolysis enlarges maximum pore-equivalent diameter by 91% within 7 days (from 80 µm to ~154 µm; Figure 1f,g), entering the ~100–200 µm range reported to support active dermal fibroblast infiltration [16,17] without post-fabrication modification; and PDO, as the slowest-absorbing component, maintains the fiber network during pore evolution. This modular logic—wherein each functional demand is independently addressable through polymer component selection—enables the scaffold architecture to evolve structurally in step with the progressive demands of tissue repair, and illustrates a design rationale that, if validated through component-specific controls in future studies, may represent a broadly applicable principle for absorbable scaffolds [7,25].

These functional outcomes are consistent with published benchmarks for the constituent materials. Regarding wettability, Faglie et al. [57] reported that 30% (*w*/*w*) poloxamer 188 incorporation into electrospun PCL fibers reduced the static contact angle from 118° to approximately 44°—a measurable but incomplete hydrophilization that still permits sessile-drop equilibration. The present composite achieved instantaneous absorption (effective *θ* ≈ 0°; Video S1), reflecting the combined effect of P188 dissolution and the water-soluble sacrificial fiber component, which eliminates residual hydrophobicity entirely. Regarding degradation, Gil-Castell et al. [15] reported that electrospun PLGA and PDO scaffolds underwent molar mass reduction to approximately 20% of initial values within 20–30 days under static hydrolytic conditions (PBS, 37 °C), with complete disintegration within 150 days. The present matrix reached Grade 5 (macroscopically undetectable) by Day 14 in vivo—faster than these acellular benchmarks, consistent with the additive contributions of cellular enzymatic activity, mechanical loading, and the inflammatory wound milieu.

### 4.2. Scaffold-Directed Cellular Accommodation: Pore Architecture Correlates with Integration

Satisfying structural criteria at the material level is necessary but insufficient; the matrix must measurably influence cellular behavior through its evolving architecture to establish functional effectiveness as a dermal scaffold. The pre-degradation experiment (Figure 3b) indicates that pore geometry—rather than culture duration or soluble factor accumulation—serves as a primary structural correlate of spreading, consistent with the evolving architecture facilitating cellular accommodation. Both L929 and BJ fibroblasts exhibited concurrent aspect ratio elevation and solidity reduction that intensified in temporal register with pore enlargement (Figure 2d–g), a morphometric signature distinguishing multi-directional exploration from uniaxial elongation [39]. This concordance across two cell lines, two analytical frameworks (categorical classification and continuous distributional morphometry), and multiple time points aligns with progressive structural evolution of the matrix translating into measurable cellular accommodation—the functional outcome that the staged-absorption design was intended to achieve. That BJ primary human fibroblasts exhibited earlier and more extensive spreading than L929 cells further suggests that the composite architecture is compatible with the migratory and contractile capacity of primary dermal fibroblasts, supporting its intended application in full-thickness wound repair. The integration of cytocompatibility, morphological, and functional characterization within a unified in vitro evaluation framework parallels recent approaches applied to multi-component scaffold systems, in which combinatorial assays were employed to resolve synergistic material–cell interactions [58]. Complementary evidence from confocal Z-stack imaging (Figure S4; Video S2) and histological cross-sections (Figure S5) further confirmed that cells are distributed within the fibrous architecture rather than confined to the material surface.

### 4.3. Accelerated Wound Repair, Timely Scaffold Absorption, and Durable Collagen Benefit

Whether in vitro cellular accommodation advantage translates to measurable healing benefit in vivo constitutes a critical preclinical question. Matrix-treated wounds demonstrated significantly accelerated wound-area reduction throughout the proliferative window (Days 7–21; Figure 4b)—the phase in which structural support is most consequential [20,59]. The matrix was macroscopically undetectable by Day 14 and elicited no adverse foreign-body reaction (ISO 10993-6), in line with fulfillment of its structural role during the inflammatory-to-proliferative transition and then cleared from the wound bed without residual material burden. The persistence of a statistically significant healing advantage at Day 21—seven days after macroscopic absorption—aligns with the prediction of the scaffold template concept articulated by Yannas et al. [6]. According to this framework, a scaffold that establishes favorable structural conditions for tissue organization becomes dispensable once the cellular process of tissue formation has been initiated. Convergence of wound area by Day 28 demonstrates that the matrix accelerates the epithelial closure trajectory rather than altering the macroscopic surface endpoint, distinguishing it from approaches that permanently modify tissue architecture. The apparently extended closure timeline relative to unsplinted models reported in the literature—in which contraction can account for 40–60% of wound closure [41,42]—is an expected consequence of the splinted design and the larger wound diameter (Ø 20 mm) employed here. As Pignet et al. [60] emphasized in their systematic evaluation of in vivo wound chronicity models, the splinted back punch model remains the current standard for evaluating regenerative interventions in rodents precisely because it minimizes contractile healing; however, residual contraction persists even with splinting, and direct comparison of absolute closure rates between splinted and unsplinted models is therefore not methodologically appropriate. Notably, while wound-area convergence at Day 28 reflects equivalent epithelial closure, quantitative collagen analysis reveals a sub-surface advantage at Day 28 (*p* < 0.01; Figure 5d)—indicating that scaffold-treated dermis retains greater collagen content at this preclinical time point. Together with the significantly greater collagen deposition during matrix residence (Day 7: *p* < 0.05), these data are compatible with the scaffold-mediated microenvironment supporting durable collagen accumulation that persists well beyond matrix absorption. To contextualize the present scaffold’s biological performance within the broader landscape of acellular electrospun systems, recent studies employing similar full-thickness wound models are considered below. Chin et al. reported that acellular wet-electrospun PCL scaffolds achieved full-thickness wound integration in rats through fibroblast migration, angiogenesis, and de novo ECM deposition without exogenous cells, confirming that scaffold architecture alone can direct regenerative outcomes in excisional models [61]. Bastidas et al. demonstrated that a bilayer PLGA/fibrin electrospun scaffold promoted granulation tissue growth and early collagen deposition in full-thickness rat wounds, with the PLGA membrane providing structural support during the proliferative phase while the hydrogel layer maintained a moist anti-inflammatory microenvironment [62].

### 4.4. Favorable Biocompatibility: Structural Facilitation Without Pharmacological Activity

A scaffold that accelerates healing through pharmacological activity or sustained inflammatory stimulation would raise distinct safety and regulatory considerations. The present data are compatible with a predominantly structural contribution of the Fibrous Matrix. Tissue IL-6 and IL-10 showed no intergroup differences at any time point (all *p* > 0.05); the ISO 10993-6 composite inflammation score, while modestly reduced in the matrix group at Day 7 (10.27 vs. 12.07; *p* = 0.0014; Table 1), converged from Day 14 onward; no foreign body giant cells or fibrous encapsulation were detected; and CD31^+^ vessel density was equivalent between groups throughout the study. This constellation—accelerated wound-area reduction without cytokine perturbation, angiogenic enhancement, or chronic inflammation—is characteristic of a biocompatible structural template that supports early tissue formation and absorbs without adverse tissue response, consistent with the requirements of an effective dermal scaffold [63]. The α-SMA/Vimentin activation ratio revealed a biphasic pattern: elevated myofibroblast activation at Day 7 (0.41 vs. 0.26) followed by decline below controls from Day 14 onward (Figure 6b). Although individual comparisons did not reach significance (*n* = 5), this trajectory is temporally consistent with the matrix’s structural timeline. This directional pattern is compatible with the following interpretation: The fibrous topography, with fibers and infiltrating cells clearly visible at Day 7 (Figure S5), provides mechanical resistance that is associated with α-SMA expression and contractile phenotype acquisition during matrix residence [21,64,65]; subsequent matrix absorption removes this stimulus, permitting earlier myofibroblast resolution [51,52]. Such a profile—efficient early contraction coupled with accelerated phenotypic resolution—is clinically favorable, as persistent myofibroblast activity is a recognized contributor to pathological scarring [21,54]. Collagen dynamics further support the structural interpretation. While the Control group exhibited the classical wound-healing collagen trajectory—accumulation peaking at Day 14 followed by substantial decline during MMP-mediated remodeling—the Fibrous Matrix group maintained stable collagen levels throughout (Day 7: *p* < 0.05; Day 28: *p* < 0.01). This pattern is consistent with scaffold-directed early fibroblast alignment promoting collagen deposition that may better resist remodeling-phase proteolysis, although direct verification would require collagen fiber orientation analysis and type-I/type-III ratio quantification. If verified, this would represent a durable structural benefit extending beyond the matrix residence period without pharmacological intervention. Systemic markers provide complementary evidence of the healing trajectory. Late-phase divergence in serum SOD activity (*p* = 0.010) and MDA concentration (*p* = 0.019) at Day 28 suggests that accelerated wound resolution may be associated with reduced cumulative oxidative burden, although a causal relationship cannot be established from the present dataset.

### 4.5. Staged Absorption as a Design Principle

The composite staged-absorption concept illustrated here extends beyond the specific tri-polymer system. The underlying principle—distributing functional demands across components with complementary absorption timescales so that architecture evolves with tissue repair—is applicable to clinical scenarios requiring temporally coordinated support [7,25]. Cartilage, tendon, and nerve repair each present analogous challenges in which initial structural guidance must eventually yield to tissue-specific organization [22,66]. The modular nature of the composite design permits systematic substitution of individual components (e.g., replacing PLGA with faster- or slower-absorbing polyesters, or incorporating bioactive moieties into specific polymer streams) to tailor the matrix’s structural evolution timeline to tissues with different repair kinetics—without altering the fabrication platform. This adaptability distinguishes the composite architecture from single-polymer systems, in which absorption rate, pore evolution, and mechanical decay are inextricably coupled. Validation of this design principle through systematic component-specific comparisons remains an important objective for future investigation.

### 4.6. Limitations and Future Directions

The splinted rat model minimizes contraction-mediated closure [41,42], but does not replicate human healing kinetics. The sole in vivo comparator was Vaseline gauze (clinically standard inert coverage); although the matrix conferred a significant wound closure advantage (Section 3.3.1), a non-degradable fibrous scaffold control would be required to attribute this benefit exclusively to the staged degradation sequence. Degradation was characterized by gravimetric and morphological (SEM) methods; molecular-level spectroscopic techniques (e.g., FTIR, NMR, or GPC) would provide direct chemical evidence of hydrolysis progression and enable molar mass tracking at intermediate time points. Mechanical evolution during degradation was not quantified; although histological evidence confirms fiber presence with cellular infiltration at Day 7 (Figure S5), fiber-scale mechanical characterization would further substantiate sustained structural support. Future investigations should additionally incorporate component-specific scaffold comparators, collagen fiber orientation analysis (e.g., second-harmonic generation imaging), systematic comparisons with commercially available dermal scaffolds, and large-animal validation. Notwithstanding these limitations, the convergent physicochemical, in vitro, and in vivo evidence supports the composite matrix as a functionally effective dermal scaffold.

## 5. Conclusions

The convergent physicochemical, cellular, and in vivo evidence presented herein functionally supports the staged-absorption design rationale. The Fibrous Matrix provides a dynamically evolving pore architecture that facilitates fibroblast accommodation, accelerates full-thickness repair throughout the healing trajectory, and is macroscopically undetectable by Day 14 without eliciting adverse foreign-body reactions—collectively satisfying the three functional criteria defining an effective dermal scaffold. Notably, the structural benefit conferred during matrix residence—durable collagen architecture—persists beyond scaffold absorption into the remodeling phase. This is consistent with scaffold-directed tissue organization being retained within the regenerating dermis and not being contingent upon continued material presence. These findings further provide preclinical evidence for staged absorption as a potential biomaterial design principle: competing functional criteria need not be compromised within a monolithic polymer when absorption timescale, rather than polymer identity, serves as the governing design variable. Products employing this composite architecture have received US FDA 510(k) clearance; prospective clinical evaluation across diverse wound etiologies will further delineate the translational scope of this platform.

## Figures and Tables

**Table 1:** Semi-quantitative inflammation scoring (H&E).

Time	Ctrl	Matrix	*p* (Matrix vs. Ctrl)
Day 7	12.07 ± 1.49	10.27 ± 1.28	0.0014 **
Day 14	10.33 ± 2.32	9.80 ± 2.14	0.52
Day 21	8.87 ± 2.17	8.13 ± 2.75	0.42
Day 28	6.53 ± 1.92	6.87 ± 1.96	0.64

Notes: Mean ± SD; *n* = 5 per group per time point. Intergroup comparisons (Matrix vs. Control) by unpaired Student’s *t*-test (balanced design). Intact skin reference: 1.47 ± 1.51 (*n* = 15); comparisons of wound groups vs. intact skin by Mann–Whitney *U* test due to unequal sample sizes and skewed baseline distribution (*p* < 0.0001 at all time points). ** *p* < 0.01.

**Table 2:** Body weight of rats during the observation period (mean ± SD, g).

Time Point	Ctrl	Fibrous Matrix	*p*
Day 0	208.58 ± 6.24	209.06 ± 5.53	>0.05
Day 3	218.54 ± 6.25	219.14 ± 4.71	>0.05
Day 7	231.80 ± 6.26	233.38 ± 6.13	>0.05
Day 14	254.51 ± 7.14	253.91 ± 6.81	>0.05
Day 21	278.38 ± 6.82	277.22 ± 6.33	>0.05
Day 28	296.37 ± 7.33	299.10 ± 7.14	>0.05

## Data Availability

The raw data supporting the conclusions of this article will be made available by the authors upon reasonable request.

## Supplementary Materials

The following supporting information can be downloaded at: https://www.mdpi.com/article/10.3390/jfb17080375/s1, Figure S1: Schematic of the electrospinning fabrication process; Figure S2: Contact angle measurement of the composite fibrous matrix; Figure S3: SEM micrographs of the composite fibrous matrix at Days 0 (a), 5 (b), 7 (c) and 14 (d) during incubation in complete culture medium (37 °C, 5% CO_2_); Figure S4: Confocal imaging of L929 fibroblasts within the composite fibrous matrix (pre-degraded, Day 1 post-seeding); Figure S5: Representative H&E-stained cross-section of the composite fibrous matrix at Day 7 post-implantation; Table S1: Morphometric analysis of L929 and BJ fibroblasts on pre-degraded matrices (7-day cell-free incubation in complete medium prior to seeding); Video S1: Wettability assessment of the composite matrix—deionized-water droplets (3–5 µL, micropipette) deposited on the matrix surface demonstrating instantaneous absorption upon contact; Video S2: Confocal Z-stack of L929 fibroblasts on composite fibrous matrix (pre-degraded, Day 1 post-seeding).
