# Supplementary material for: In Vitro and In Vivo Evaluation of a Composite Electrospun Matrix as a Dermal Scaffold: Cell Behavior and Full-Thickness Wound Repair
Source: J Funct Biomater. 2026 Aug 1;17(8):375. doi: 10.3390/jfb17080375 (PMC13514176; doi:10.3390/jfb17080375)
Supplement: Supplementary file 1 [file jfb-17-00375-s001.zip › Supplementary Materials.pdf]

Supplementary Materials

In Vitro and In Vivo Evaluation of a Composite Electrospun Matrix as a Dermal Scaffold: Cell Behavior and Full-Thickness Wound Repair

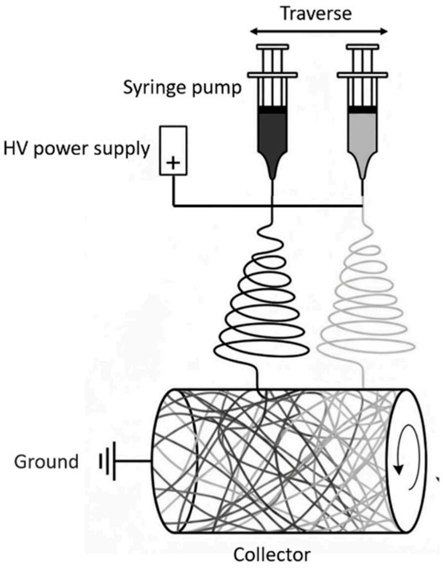

Figure S1. Schematic of the electrospinning fabrication process.

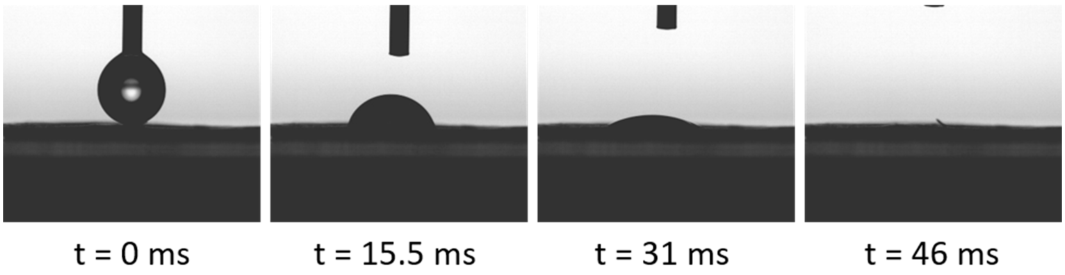

Figure S2. Contact angle measurement of the composite fibrous matrix. Water droplet (~3 μL) deposited via goniometer; complete absorption within ~46 ms (effective $\theta \approx 0°$). Frames at t = 0, 15.5, 31, and 46 ms.

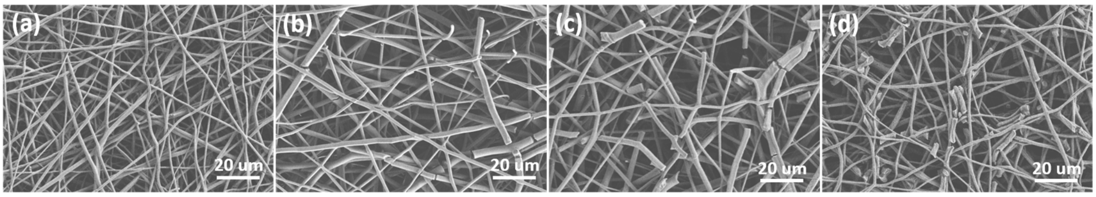

Figure S3. SEM micrographs of the composite fibrous matrix at Days 0 (a), 5 (b), 7 (c) and 14 (d) during incubation in complete culture medium (37 °C, 5% CO_2). Scale bars = 20 µm.

Table S1. Morphometric analysis of L929 and BJ fibroblasts on pre-degraded matrices (7-day cell-free incubation in complete medium prior to seeding). Analysis performed as described in Section 2.2.7.

(a) L929 morphological classification—pre-degraded matrices

**Day**	**AR (mean ± SD)**	**Solidity (mean ± SD)**	**Round (%)**	**Spindle (%)**	**Stellate (%)**
**1**	1.295 ± 0.057	0.924 ± 0.010	54.6	3.8	13.8
**7**	1.578 ± 0.058	0.921 ± 0.001	34.5	16.5	6.0

(b) BJ continuous morphometrics—pre-degraded matrices

**Day**	**AR (mean ± SD)**	**Solidity (mean ± SD)**	**EI (mean ± SD)**
**1**	3.57 ± 0.74	0.613 ± 0.091	0.463 ± 0.078

(c) Statistical comparisons (Mann–Whitney *U* test, single-cell level):

**Comparison**	**AR**	**Solidity**	**Circularity**
L929 Pre-deg D1 vs Fresh D1	$p < 0.001$	$p < 0.001$	$p < 0.001$
L929 Pre-deg D1 vs Fresh D3	$p < 0.001$	$p < 0.001$	$p < 0.001$
L929 Pre-deg D7 vs Fresh D7	n.s.	n.s.	n.s.
BJ Pre-deg D1 vs Fresh D3	—	$p = 0.019$	—

Note: Mann–Whitney *U* tests at the single-cell level (BJ: $n \geq 130$ per group; L929: $n \geq 500$ per group).

L929 Pre-deg Day 1 metrics were significantly shifted from Fresh Day 1 toward Fresh Day 3 in all descriptors (all $p < 0.001$). The residual significance of the Pre-deg D1 vs Fresh D3 comparison is consistent with the high statistical power inherent to large single-cell sample sizes rather than a large biological effect; category distributions (Round: 54.6% vs >60% at Fresh D1; Figure 2b) confirm that Pre-deg D1 is directionally closer to Fresh D3 than to Fresh D1, supporting accelerated

accommodation by approximately 2 days. BJ Pre-deg D1 Solidity was significantly lower than Fresh D3, indicating spreading at or beyond Fresh D3 levels. By Day 7, L929 on pre-degraded matrices were morphologically indistinguishable from Fresh Day 7 (all $p > 0.05$), attributable to progressive cell integration within the fiber network limiting the resolution of surface-based morphometric analysis.

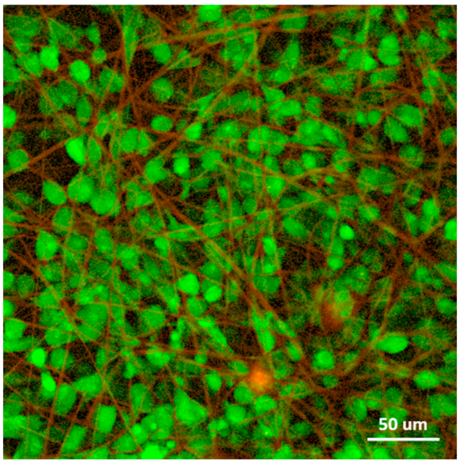

Figure S4. Confocal imaging of L929 fibroblasts within the composite fibrous matrix (pre-degraded, Day 1 post-seeding). Representative optical section from Z-stack (30 slices, step 1.06 μm). Green: live cells (calcein-AM); Red: scaffold fiber. Cells interspersed among fibers, confirming cellular accommodation within the matrix architecture. Scale bar = 50 μm.

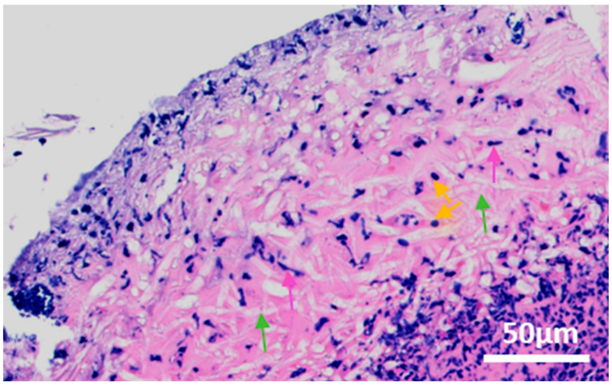

Figure S5. Representative H&E-stained cross-section of the composite fibrous matrix at Day 7 post-implantation. Green arrows: scaffold fibers; Pink arrows: fibroblast-like cells; Yellow arrows: neutrophils. Cells are observed distributed among scaffold fibers. Scale bar = 50 μm.

Video S1: Wettability assessment of the composite matrix—deionized-water droplets (3–5 μL, micropipette) deposited on the matrix surface demonstrating instantaneous absorption upon contact.

Video S2: Confocal Z-stack of L929 fibroblasts on composite fibrous matrix (pre-degraded, Day 1 post-seeding). 30 slices; step size: 1.06 μm. Green: live cells (calcein-AM); Red: scaffold fibers.
